# A model for predicting both breast cancer risk and non-breast cancer death among women > 55 years old

**DOI:** 10.1186/s13058-023-01605-8

**Published:** 2023-01-24

**Authors:** Mara A. Schonberg, Emily A. Wolfson, A. Heather Eliassen, Kimberly A. Bertrand, Yurii B. Shvetsov, Bernard A. Rosner, Julie R. Palmer, Long H. Ngo

**Affiliations:** 1 Division of General Medicine and Primary Care, Department of Medicine, Harvard Medical School, Beth Israel Deaconess Medical Center, Boston, MA USA; 2 Department of Epidemiology, Harvard School of Public Health, Boston, MA USA; 3 Channing Division of Network Medicine, Brigham and Women’s Hospital, Harvard Medical School, Harvard School of Public Health, Boston, MA USA; 4 Slone Epidemiology Center, Boston University, Boston University School of Medicine, Boston, MA USA; 5 University of Hawaii Cancer Center, University of Hawaii at Manoa, Manoa, HI USA

**Keywords:** Mortality prediction, Breast cancer prediction, Competing risks

## Abstract

**Background:**

Guidelines recommend shared decision making (SDM) for mammography screening for women ≥ 75 and not screening women with < 10-year life expectancy. High-quality SDM requires consideration of women’s breast cancer (BC) risk, life expectancy, and values but is hard to implement because no models simultaneously estimate older women’s individualized BC risk and life expectancy.

**Methods:**

Using competing risk regression and data from 83,330 women > 55 years who completed the 2004 Nurses’ Health Study (NHS) questionnaire, we developed (in 2/3 of the cohort, *n* = 55,533) a model to predict 10-year non-breast cancer (BC) death. We considered 60 mortality risk factors and used best-subsets regression, the Akaike information criterion, and c-index, to identify the best-fitting model. We examined model performance in the remaining 1/3 of the NHS cohort (*n* = 27,777) and among 17,380 Black Women’s Health Study (BWHS) participants, ≥ 55 years, who completed the 2009 questionnaire. We then included the identified mortality predictors in a previously developed competing risk BC prediction model and examined model performance for predicting BC risk.

**Results:**

Mean age of NHS development cohort participants was 70.1 years (± 7.0); over 10 years, 3.1% developed BC, 0.3% died of BC, and 20.1% died of other causes; NHS validation cohort participants were similar. BWHS participants were younger (mean age 63.7 years [± 6.7]); over 10-years 3.1% developed BC, 0.4% died of BC, and 11.1% died of other causes. The final non-BC death prediction model included 21 variables (age; body mass index [BMI]; physical function [3 measures]; comorbidities [12]; alcohol; smoking; age at menopause; and mammography use). The final BC prediction model included age, BMI, alcohol and hormone use, family history, age at menopause, age at first birth/parity, and breast biopsy history. When risk factor regression coefficients were applied in the validation cohorts, the c-index for predicting 10-year non-BC death was 0.790 (0.784–0.796) in NHS and 0.768 (0.757–0.780) in BWHS; for predicting 5-year BC risk, the c-index was 0.612 (0.538–0.641) in NHS and 0.573 (0.536–0.611) in BWHS.

**Conclusions:**

We developed and validated a novel competing-risk model that predicts 10-year non-BC death and 5-year BC risk. Model risk estimates may help inform SDM around mammography screening.

**Supplementary Information:**

The online version contains supplementary material available at 10.1186/s13058-023-01605-8.

## Introduction

Breast cancer is the most common non-skin cancer diagnosed in women and incidence increases with age [1, 2]. Mammography screening reduces breast cancer mortality by 19% in women 40–74 years. However, there is a delay in benefit; on average it takes 10.7 years for 1 in 1000 women screened to avoid breast cancer death [3, 4]. There are also harms to screening, including anxiety, complications from workup of cancer, and overdiagnosis (detection of non-lethal tumors) [5]. Therefore, guidelines recommend not screening women who have a life expectancy of less than 10 years. [6–8] Despite these recommendations, 40–55% of community dwelling US women ≥ 65 years with < 10 year life expectancy are screened; most are at low or average breast cancer risk. [9, 10]

In addition, none of the mammography screening trials included women ≥ 75 years old. Most guidelines recommend engaging women ≥ 75 in shared decision making [7, 8, 11, 12]. High-quality shared decision making around mammography screening requires consideration of breast cancer risk, life expectancy, and values and preferences [5]. However, shared decision making rarely occurs and many older women overestimate their breast cancer risk and screening’s benefits [13–15]. Furthermore, while guidelines recommend biennial screening for women ≥ 55 years, many older women choose to be screened annually; personalized information about breast cancer risk may help these women decide how often to be screened [16, 17]. Despite the need, there are no tools that simultaneously estimate older women’s individualized breast cancer risk *and* life expectancy to support shared decision making around mammography screening.

The Breast Cancer Risk Assessment Tool (BCRAT, a.k.a. “Gail Model”) is the most commonly used breast cancer prediction model in primary care [18, 19]. It considers a woman’s age, age at menarche, age at first birth, history of breast biopsy (including presence of atypia), breast cancer family history and race/ethnicity to estimate 5-year breast cancer risk for women up to age 85. We previously examined BCRAT’s performance in in the Nurses’ Health Study (NHS) and Women’s Health Initiative and found that BCRAT overestimated 5-year breast cancer risk by 5–20% in women ≥ 55 years and by 10–30% in women ≥ 75 and had modest discrimination (c-statistic 0.57–0.58) [20]. We hypothesized that BCRAT overestimated breast cancer risk in older women because while it accounts for age-based risk of non-breast cancer (non-BC) death in estimating breast cancer risk, it does not account for women’s individualized non-BC death risk. Therefore, we aimed to develop a novel model that would simultaneously predict breast cancer risk and non-BC death in women ≥ 55 years.

We previously used NHS data and Fine-Gray competing risk regression to develop a breast cancer prediction model for older women [21]. That model included women’s age, family history of breast cancer, reproductive factors, health behaviors, and prior mammography use (because screening increases breast cancer detection and may confound the influence of some risk factors on breast cancer incidence) [22]. It also included six mortality risk factors (history of stroke, diabetes, myocardial infarction, emphysema, heart failure, and limitation in performing moderate activity) that were added based on expert opinion to apply weights to women’s probability of a competing non-BC death when estimating their breast cancer risk. Our current aim was to develop and validate a non-BC death prediction model and then include predictors from this model in our competing risk breast cancer risk prediction model.

## Methods

We used NHS data to extend our previously developed competing risk breast cancer prediction model to also predict non-BC death [23]. NHS is a longitudinal study of 121,738 female nurses aged 30–55 years at entry in 1976; 97% who were white. Since Black women are more likely to die of breast cancer and be diagnosed at earlier ages than white women, we further examined model performance in the Black Women’s Health Study (BWHS) [24, 25]; a longitudinal study of 59,000 self-identified Black women ages 21–69 at entry in 1995. At baseline and in biennial follow-ups, participants in both cohorts provide detailed lifestyle and medical history information through mailed questionnaires (Additional file 1: Appendix A provides additional details about each cohort). Our study samples (*n* = 83,330 NHS, *n* = 17,380 BWHS; see Additional file 1: eFigure 1) included postmenopausal women without a history of invasive or noninvasive breast cancer who returned the 2004 NHS questionnaire (could be returned through May 2006) or 2009 BWHS questionnaire (96.3% who returned  the questionnaire returned it by the end of 2010). We chose the 2004 NHS questionnaire for study initiation since: (1) similar to current practice most women had stopped using menopausal hormone therapy (MHT); (2) it included functional assessments; and (3) it allowed > 10 years follow-up. We chose the 2009 BWHS questionnaire for study initiation since it allowed 10 years follow-up for most women and allowed a maximum number of BWHS participants to be included (i.e., to have reached age 55). NHS participants were 57–85 years, and BWHS participants were 55–85 at study entry. The study was approved by the institutional review boards of Boston University Medical Center, Brigham and Women’s Hospital, Harvard T.H. Chan School of Public Health, and those of participating registries as required.

### Outcomes

The oldest participants at the end of follow-up were aged 95. In both cohorts, cause of death was determined from state-issued death certificates, the National Death Index, family and friends, and the post-office. In NHS, death information was further supplemented with medical record review; > 98% of deaths are identified [26, 27]. For NHS, we included breast cancers confirmed by medical record review (88%) or self-reported (12%) since validation studies have found self-reported breast cancers in NHS to be accurate [28]. In BWHS, breast cancers are identified through self-report or through 24 state cancer registries (> 95% of BWHS participants live in these states) and are confirmed by review of hospital and state cancer registry pathology records (> 99% are confirmed) [29, 30]. We excluded women with a history of cancer (except non-melanomatous skin cancer) since NHS did not consistently confirm second cancer diagnoses.

### Mortality risk factors

To expand our model to predict non-BC death, we considered 60 potential mortality risk factors, including health behaviors (4), comorbidity (32), physical function (16), psychosocial factors (5), age, age at menopause, and parental longevity; information was obtained from the 2004 NHS questionnaire and/or prior years (see Additional file 1: Appendix B for variable definitions). We only included factors that may be self-reported (e.g., no laboratory values) for ease of clinical implementation; however, in sensitivity analyses, we repeated our analyses using confirmed diseases when available. We did not consider socioeconomic factors (e.g., income) because once the model is implemented we do not want women to be denied screening because of a low estimated life expectancy based on socioeconomic status.

### Breast cancer risk factors

After identifying the best-fitting model for non-BC death, we then re-examined our model’s performance in predicting breast cancer including the risk factors identified for non-BC death [21]. For these analyses, we censored women with noninvasive breast cancer or other cancers at the time of diagnosis. We also re-examined model performance including risk factors as continuous rather than categorical variables if linearly associated with breast cancer risk. Since measured mammographic density was only available for 2174 NHS participants, we used predicted mammographic density as performed previously by Rice et al. (predicted and actual mammographic density are correlated [Spearman correlation of 0.61]). The validated mammographic density prediction model considers age, current BMI, BMI at age 18, adolescent somatotype, parity, age at first birth, postmenopausal status, alcohol use, benign breast disease, and MHT use. [31]

### Non-BC death model development

Analyses were completed using SAS 9.4 software. We randomly divided the NHS population into 2/3 (*n* = 55,553) for model development and 1/3 (*n* = 27,777) for internal validation. Survival time was measured from study entry until non-BC death; participants were censored at breast cancer death or 10 years from their 2004 questionnaire return date, whichever came first. We first examined the unadjusted effect of each mortality risk factor using proportional hazards regression (PHR). Variables significantly associated (*p* < 0.05) with non-BC death in univariate analyses and not collinear at > 0.4 (Spearman correlation) were considered in our multivariable model. When two variables were collinear, we removed the variable more difficult to self-report. We used best-subsets regression (allowing comparison of all possible models and selecting those with the highest global score chi-square statistic) [31], the Akaike information criterion (AIC, a function of the log-likelihood that adds a penalty of 2 for each additional factor; lower AICs indicate better fit), and the c-index (estimate of area under the receiver operating characteristic curve) to identify the best-fitting models for non-BC death [32–34]. Investigators reviewed the top models associated with the highest c-index and lowest AIC to select the best model. The proportional hazards assumption was evaluated by computing Schoenfeld residuals and visually examining log–log survival curves; no apparent violations were identified. Since few methods exist for covariate selection using competing risk regression and breast cancer death is a rare competing risk to non-BC death, we hypothesized that using cause-specific PHR for covariate selection would identify the same top models as competing risk regression. To confirm, we reviewed the AIC and c-index of the 10 best-fitting models and found that the AICs and c-indices were similar using either method. We determined the subdistribution hazard ratio (HR) for each risk factor in our final model using competing risk regression and computed cause-specific cumulative incidence functions (CIFs) for breast cancer death and non-BC death.

In sensitivity analyses, we examined for “ghost-time” bias (to examine the potential effect of including data from individuals who may have died but not yet captured) by censoring participants at age 90 [35]. We also calculated age-adjusted c-indices, used multiple imputation to impute missing data (see Additional file 1: Appendix C for multiple imputation details). In addition, we compared our new model’s performance in predicting non-BC death to a model that included only the 6 mortality risk factors chosen by expert opinion to predict the competing risk of non-breast cancer death in our original breast cancer prediction model.

### Internal and external validation

We examined the final model’s performance in predicting 10-year non-BC death and 5-year breast cancer risk because these thresholds have clinical significance. Guidelines for use of breast cancer prevention medications consider postmenopausal women with ≥ 3% 5-year risk to be at high risk [36, 37]. Also, prior studies have shown that individuals with ≥ 50% 10-year mortality risk tend to have < 10-year life expectancy since life expectancy is the median survival of a population [38, 39].

We used Royston and Altman’s methods for validating models using survival analyses and examined our model’s calibration (whether model predicted probabilities are accurate) and discrimination (how well our model distinguishes between individuals who do and do not develop an outcome) in predicting non-BC death [40, 41]. First, we compared the prevalence and regression coefficients associated with each risk factor in the development and validation cohorts using normal approximation *z*-tests. While most risk factors were defined similarly by NHS and BWHS, BWHS did not assess participant mobility or ability to bath/dress oneself. We censored BWHS participants without complete 10-year follow-up on December 31, 2020, since death data after that date may have been incomplete.

Calibration of the model in predicting non-BC death was assessed by estimating the ratio of the expected survival (1-CIF for non-BC death from our competing risk regression model) to the observed survival (1-the observed CIF computed using the nonparametric estimation of CIF) at 5 and 10 years within risk quintiles [42]. To test discrimination, we calculated the model’s c-index in the validation cohorts using risk factor regression coefficients from the development cohort using Kremer’s SAS macro [43] based on the work of Harrell et al. [44] and Pencina et al. [45]. Additional file 1: Appendix C provides additional details on methods used for model validation. We repeated these methods to examine model performance in predicting non-BC death by age (55–74, 75+) and in predicting breast cancer risk overall and by age.

### Examples

To demonstrate how our model may be useful, we calculated breast cancer and non-BC death risk estimates for four example women 75 years old for whom guidelines recommend shared decision making and to not screen women with < 10 year life expectancy [7, 8, 11]. We also presented the proportion of women in our validation cohorts who would be estimated to be at higher or lower risk of non-BC death (using a 50% 10-year mortality risk threshold) and of breast cancer (using a 3% 5-year breast cancer risk threshold) based on model risk estimates.

## Results

NHS development cohort participants (*n* = 55,553) were 96.2% non-Hispanic white, and their mean age was 70.1 (SD 7.0) years. Over 10 years, 3.1% developed breast cancer, 0.3% died of breast cancer, and 20.1% died of other causes. NHS validation cohort (*n* = 27,777) participants were similar to development cohort participants (≤ 0.5% difference for any characteristic, Table 1). BWHS participants (*n* = 17,380) differed by race, were younger, more likely to have had a mammogram, a breast biopsy, have higher BMI, younger age at menopause, comorbidity, to be nulliparous and to walk briskly than NHS development cohort participants; BWHS participants were less likely to use alcohol, cigarettes, or MHT. The number of breast cancer diagnoses was similar between cohorts, but BWHS participants were slightly more likely to die of breast cancer; after standardizing by age the cohorts had similar rates of non-BC death. Additional file 1: eTable 1 demonstrates differences across cohorts in participant characteristics by age group (55–74, 75+ years).

**Table 1:** Participant baseline characteristics in the NHS development and validation cohorts and in BWHS

	NHS development cohort	NHS validation cohort^a^	BWHS (Crude)	BWHS (Age-standardized)^a^
N	55,553	27,777	17,380	17,380
Factors in our final model				
Age, mean (SD)	70.1 (7.0)	70.2 (7.0)	63.7 (6.7)	–
55–59 years, %	7.1	6.7	36.5	–
60–64 years, %	21.4	21.5	28.3	–
65–69 years, %	22.4	22.3	17.1	–
70–74 years, %	20.9	20.8	9.9	–
75–79 years, %	18.1	18.8	5.8	–
80+ years, %	10.1	10.0	2.4	–
Highest self-reported Body Mass Index (BMI) in past 10 years kg/m^2^, mean (SD)	28.1 (5.7)	28.1 (5.8)	32.1 (7.0)	31.6 (6.5)
Highest self-reported BMI in past 10 years				
< 20 kg/m^2^, %	2.7	2.7	0.5	0.5
20–22.4 kg/m^2^, %	10.9	11.2	3.0	3.0
22.5–24.9 kg/m^2^, %	19.1	19.4	8.5	9.2
25–29.9 kg/m^2^, %	36.5	36.3	31.6	33.7
30–34.9 kg/m^2^, %	19.5	18.9	28.3	28.0
35–39.9 kg/m^2^, %	7.1	7.3	14.5	13.5
40+ kg/m^2^, %	4.1	4.1	12.6	10.7
Unknown, %	0.1	0.2	1.0	1.3
Average alcohol use per day (highest average use in past 10 years)^b^				
None, %	37.0	37.0	50.4	54.1
1–4.9 g/day, %	22.5	22.7	26.0	23.9
5–14.9 g/day, %	17.5	17.1	13.7	12.6
15+ g/day, %	13.1	13.4	9.9	9.3
Unknown, %	10.0	9.8	–	–
Cigarette use				
Never	44.7	44.8	53.4	50.5
Current	7.7	7.9	10.1	8.0
Past	47.4	47.1	36.4	41.5
Unknown	0.2	0.2	–	–
Limited from walking several blocks^c^				
Not at all	61.8	61.5	–	–
A little or a lot	32.8	33.1	–	–
Unknown, %	5.4	5.4	–	–
Limited in bathing or dressing oneself^c^				
Not at all	88.2	87.9	–	–
A little or a lot	6.5	6.8	–	–
Unknown, %	5.3	5.3	–	–
Usual walking pace outdoors				
Unable to walk^c^	2.6	2.8	–	–
Slow or average (less than 3 mph)	73.1	72.6	62.0	65.1
Brisk/very brisk (≥ 3 mph)	19.7	20.0	27.5	23.3
Unknown, %	4.6	4.6	10.6	11.6
High Blood pressure^d^	60.3	60.3	68.7	75.4
Depression	20.4	20.5	22.2	19.4
Hip fracture	2.1	2.2	0.8	1.1
Parkinson’s disease	0.7	0.6	0.2	0.3
Myocardial infarction	5.5	5.7	4.3	6.5
Congestive heart failure	3.3	3.4	3.2	4.6
Stroke/transient ischemic attack	7.5	7.5	4.2	5.7
Emphysema/Asthma	18.6	18.5	18.3	18.0
Diabetes	12.2	11.7	24.2	28.5
Dementia	1.2	1.2	0.2	0.5
Kidney disease	0.5	0.6	1.9	2.3
Cancer (excluding non-melanomatous skin cancers)	12.3	12.2	5.7	7.4
Age at menopause (years)				
< 45, %	10.8	10.4	19.3	21.2
45–49, %	23.4	23.9	20.1	19.2
50–54, %	56.1	56.2	27.4	25.9
55+, %	8.8	8.5	8.3	10.6
Hysterectomy, age unknown, %^e^	–	–	22.2	21.9
Unknown, %	1.0	1.0	2.7	1.2
Mammogram in past 2 years^f^				
No	12.1	12.2	14.0	15.2
Yes	79.4	79.5	86.0	84.8
Unknown	8.5	8.3	–	–
Number of breast biopsies				
0, %	73.2	73.2	67.0	65.7
1, %	23.5	23.6	21.3	21.3
2+, %	3.3	3.2	11.7	13.1
Postmenopausal hormone use				
Never, %	22.5	22.9	40.7	37.5
Current estrogen plus progestin user < 5 years, %	0.4	0.5	0.7	0.2
Current estrogen plus progestin user 5 + years, %	2.7	2.7	1.1	0.8
Current estrogen-alone user < 5 years, %	0.9	0.8	1.6	0.7
Current estrogen-alone user 5 + years, %	9.4	9.1	6.3	5.6
Past estrogen plus progestin user < 5 years, %	14.9	14.9	12.8	12.5
Past estrogen plus progestin user 5 + years, %	15.8	16.1	5.6	7.4
Past estrogen-alone user < 5 years, %	7.1	7.2	12.2	12.2
Past estrogen-alone user 5 + years, %	13.8	13.8	13.8	18.1
Unknown, %	12.6	12.2	5.3	4.8
Age at first birth (years) and parity^g^				
Nulliparous	5.3	5.4	16.0	12.7
< 25, 1–2 children	14.2	13.9	32.2	35.4
< 25, 3+ children	35.2	34.9	27.8	28.3
25–29, 1–2 children	14.8	15.3	11.9	10.8
25–29, 3+ children	20.1	20.3	2.9	4.0
30+, 1–2 children	5.8	5.8	7.1	6.3
30+, 3+ children	2.8	2.8	0.5	0.6
Unknown	1.8	1.7	1.6	1.9
Number of first-degree relatives with history of breast cancer and age at diagnosis^h^				
None, %	82.1	81.9	80.1	79.8
1 and age < 50, %	4.3	4.1	3.8	4.2
1 and age 50+, %	11.6	11.8	14.2	14.0
2+ and at least one age < 50, %	1.0	1.1	0.6	0.7
2+ and age 50+, %	1.1	1.1	1.3	1.3
Outcomes				
Breast cancer in 5-year follow-up, %	1.7	1.7	1.7	1.8
Breast cancer in 10-year follow-up, %	3.1	3.2	3.1	3.2
Breast cancer death in 5-year follow-up, %	0.1	0.1	0.2	0.2
Breast cancer death in 10-year follow-up, %	0.3	0.2	0.4	0.6
Non-breast cancer death 5-year follow-up, %	7.7	8.0	4.5	8.1
Non-breast cancer death 10-year follow-up, %	20.1	20.6	11.1	19.7
Not included in model				
Race/ethnicity				
Non-Hispanic White, %	96.2	96.3	0.0	0.0
Non-Hispanic Black, %	1.8	1.6	99.1	99.9
Hispanic,%	0.9	0.9	0.9	0.1
Asian, Pacific Islander %	0.8	0.9	0.0	0.0
Native American, %	0.2	0.2	0.0	0.0
Education (years)^i^				
< 12	–	–	2.7	4.0
12	–	–	16.4	19.1
13–15	–	–	27.8	26.2
16	91.7	91.7	19.5	17.1
17+	8.4	8.4	33.6	33.5
Unknown	0.0	0.0	0.1	0.1
Predicted mammographic density^j^	*n* = 35,073	*n* = 17,482		
Higher than median (≥ 24.7% dense)	50.0	50.1	–	–
Lower than median (< 24.7% dense)	50.0	49.9	–	–

NHS (Nurses’ Health Study) included participants that completed the 2004 questionnaire. BWHS (Black Women’s Health Study) included participants that completed the 2009 questionnaire

^a^There were no significant differences between the NHS development and validation cohorts, except for limited bathing/dressing (*p* = 0.04) and diabetes (*p* = 0.04). All comparisons between NHS development cohort and the BWHS age-standardized cohort were statistically significant except for emphysema/asthma, 5- and 10-year breast cancer incidence, and 5- and 10-year non-breast cancer death incidence. To calculate the age-standardized values for BWHS, we weighted each of six 5-year age groups, where the weight corresponded to the proportion of that age group in the whole NHS sample, and then summed the weighted age-specific values to get the overall adjusted prevalence

^b^A standard drink is any drink that contains about 14 g of pure alcohol (12 oz. of beer, 5 oz. of wine or 1.5 oz. of liquor)

^c^BWHS does not ask about participants’ about walking several blocks, limitations in bathing or dressing oneself, or inability to walk

^d^Health conditions were self-reported

^e^Participants with hysterectomy and age at menopause unknown in BWHS were placed in the 45–49 years category for all analyses; sensitivity analyses were performed showing no significant difference when placed in any other age category

^f^Participants missing data on mammography use in NHS had completed a short version of the 2004 questionnaire

^g^Participants who were parous with an unknown number of children were categorized as having 1–2 children

^h^Relatives with an unknown age at diagnosis were categorized as having been diagnosed at 50 + years of age

^i^Participants in NHS who had a master’s or doctorate degree were placed in the 17 + years category. All others were placed in the 16 years category, as registered nurses, (22.8% of these specifically reported a Bachelor’s degree)

^j^Predicted mammographic density was defined using a 9-item validated index. Prevalence is reported for those with complete data for the prediction model [18]

### Predicting non-BC death

Additional file 1: eTable 2 includes all 60 variables considered in predicting non-BC death and the reasons certain variables were removed. Best-subsets regression resulted in 961 top models; 281 had the highest c-index of 0.789. Within this group, the AIC varied by < 0.02%. Based on clinical judgment, effect on model performance, and ease of self-report, we included the 20 variables (age, BMI, alcohol use, cigarette use, function, mobility, walking pace, age at menopause, and 12 diseases) that made it into > 97% of the top 281 models; the other variables made it into < 84% of these models. Since mammography use in the past two years predicts breast cancer death (the competing risk of non-BC death), we included it in the model when predicting non-BC death. Using competing risk regression, the model’s c-index was 0.795 (0.791–0.800) for predicting 10-year non-BC death in the development cohort (Table 2), which is higher than the c-index (0.778 [0.773–0.782]) of the model when only including the 6 mortality risk factors previously selected based on expert opinion for our competing risk breast cancer prediction model.

**Table 2:** Final model for predicting non-breast cancer death in the NHS cohorts and in BWHS

Predicting non-breast cancer death	NHS development cohort		NHS validation cohort		BWHS	
Outcome being predicted	10 years	*P* value	10 years	*P* value	10 years	*P* value
	HR		HR		HR	
	n = 48,102		*n* = 24,088		*n* = 15,001	
N for that outcome	9376		4790		1592	
Factors in our final model						
Age (per year increase)	1.11	< 0.001	1.11	< 0.001	1.10	< 0.001
Highest self-reported Body Mass Index (BMI) in past 10 years: < 20 kg/m^2^	1.63	< 0.001	1.57	< 0.001	2.71	< 0.001
20–22.4 kg/m^2^	1.15	< 0.001	1.10	0.08	1.21	0.25
22.4–24.9 kg/m^2^	1	–	1	–	1	–
25–29.9 kg/m^2^	0.91	0.003	0.87	< 0.001	0.85	0.09
30–34.9 kg/m^2^	0.88	< 0.001	0.80	< 0.001	0.87	0.17
35–39.9 kg/m^2^	0.91	0.07	0.93	0.28	0.98	0.87
40+ kg/m^2^	1.10	0.10	0.97	0.70	1.28	0.03
Average alcohol use per day (highest average use in past 10 years): None	1	–	1	–	1	–
1–4.9 g/day	0.86	< 0.001	0.94	0.13	0.92	0.20
5–14.9 g/day	0.91	0.004	0.90	0.02	0.97	0.71
15+ g/day	1.04	0.24	0.96	0.43	1.28	0.004
Cigarette use: Never	1	–	1	–	1	–
Current	2.46	< 0.001	2.34	< 0.001	2.66	< 0.001
Past	1.35	< 0.001	1.30	< 0.001	1.39	< 0.001
Limited from walking several blocks:						
Not at all	1	–	1	–	–	–
A little/a lot	1.58	< 0.001	1.53	< 0.001	–	–
Limited in bathing/dressing oneself:						
Not at all	1	–	1	–	–	–
A little or a lot	1.47	< 0.001	1.48	< 0.001	-	–
Walking pace: Unable to walk	1.42	< 0.001	1.40	< 0.001	-	–
Slow or average (< 3 mph)	1	–	1	–	1	–
Brisk/very brisk (≥ 3 mph)	0.69	< 0.001	0.74	< 0.001	0.72	< 0.001
High Blood pressure	1.15	< 0.001	1.21	< 0.001	1.15	0.04
Depression	1.17	< 0.001	1.16	< 0.001	1.10	0.14
Hip fracture	1.31	< 0.001	1.31	< 0.001	1.29	0.23
Parkinson’s disease	2.35	< 0.001	2.32	< 0.001	4.09	< 0.001
Myocardial infarction	1.27	< 0.001	1.16	0.005	1.10	0.32
Congestive heart failure	1.76	< 0.001	1.97	< 0.001	2.12	< 0.001
Stroke/transient ischemic attack	1.19	< 0.001	1.24	< 0.001	1.58	< 0.001
Emphysema/Asthma	1.29	< 0.001	1.27	< 0.001	1.03	0.68
Diabetes	1.36	< 0.001	1.31	< 0.001	1.46	< 0.001
Dementia	3.07	< 0.001	2.88	< 0.001	1.79	0.04
Kidney disease	1.28	0.07	1.28	0.22	2.31	< 0.001
Cancer*	1.45	< 0.001	1.44	< 0.001	2.60	< 0.001
Age at menopause (years), < 45	1.01	0.74	0.95	0.39	1.00	0.96
45–49	1	–	1	–	1	–
50–54	0.93	0.004	0.91	0.007	0.91	0.15
55+	0.87	0.002	0.86	0.02	0.97	0.72
Mammogram in past 2 years|| No	1	–	1	–	1	–
Yes	0.75	< 0.001	0.74	< 0.001	0.61	< 0.001
C-index (95% CI)	0.795 (0.791–0.800)		0.790 (0.784–0.796)		0.780 (0.768–0.791)	
C-index (95% CI) when using risk factor regression coefficients from the NHS development cohort in the NHS and BWHS validation cohorts	–		0.790 (0.784–0.796)		0.768 (0.757–0.780)	

* Significance was defined as a *p*-value of <0.05

NHS = Nurses’ Health Study, BWHS = Black Women’s Health Study

Neither censoring follow-up of participants at age 90 nor using confirmed (only available for stroke and myocardial infarction) rather than self-reported diagnoses changed the model’s c-index. Adjusting model c-indices for age led to a small decrease in model performance (Additional file 1: eTable 3). The model’s c-index was the same using multiple imputation or a complete case analysis, and the HRs for all predictors were within 8% of each other except for kidney disease (17% difference in HRs) which was the rarest disease (0.6% prevalence, Additional file 1: eTable 4). Therefore, our final model included women with complete data. Regardless of whether competing risk regression or cause-specific PHR was used, the model’s performance was similar (PHR c-index = 0.796 [0.791–0.800] for predicting 10-year non-BC death) and risk factor hazard ratios (HRs) were within 3% of each other (Additional file 1: eTable 5).

### Internal and external validation

**Fig. 1:**
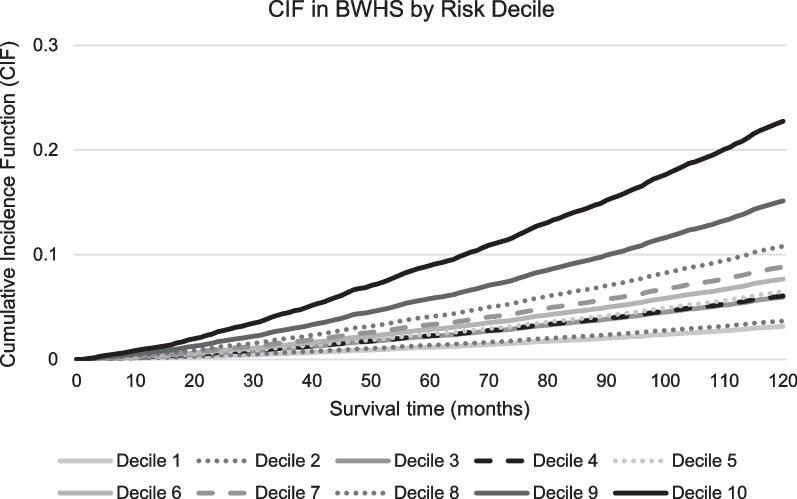
Non-BC death cumulative incidence function (CIF) curves in the BWHS over time by 10-year risk deciles

Although most risk factor HRs for predicting non-BC death differed significantly between the development cohort and the validation cohorts, likely due to large cohort sample sizes, directions of the associations were similar (Table 2). When the non-BC death model was applied to the validation cohorts, c-indices were 0.790 (0.784–0.796) in the NHS validation cohort and 0.768 (0.757–0.780) in the BWHS. Figure 1 presents non-BC death survival curves in BWHS over time by 10-year risk deciles. The c-index for prediction of 10-year non-BC death in women 55–74 was 0.760 (0.749–0.770) in the NHS validation cohort and 0.735 (0.721–0.750) in BWHS; among women ≥ 75 the c-indexes were 0.696 (0.686–0.706) and 0.671 (0.645–0.696) in NHS and BWHS, respectively (Additional file 1: eTable 6).

**Fig. 2:**
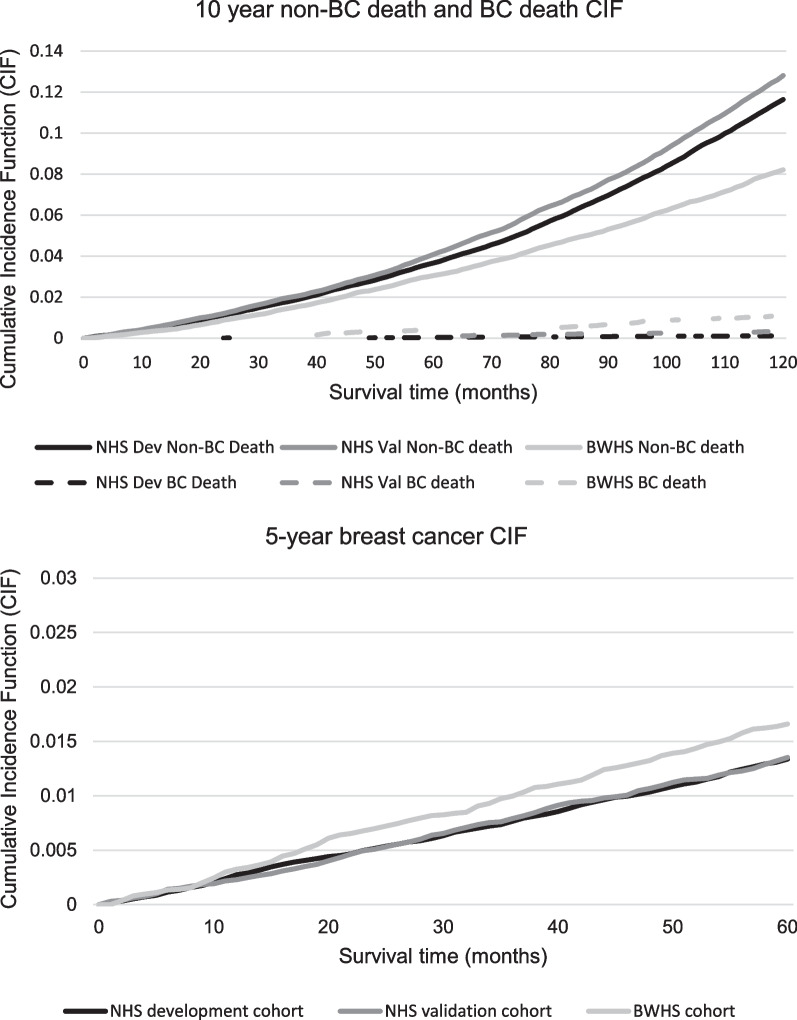
CIFs for 10-year non-BC and breast cancer death and 5-year breast cancer from each cohort. Our competing risk model for predicting non-BC death yielded two CIF functions, one for the outcome of interest of non-BC death and one for breast cancer death

Figure 2 demonstrates the CIF for 10-year non-BC death in each cohort, and Table 3 demonstrates how the expected-to-observed ratio of predicted risk approached 1 for each risk quintile for each outcome; except for women in the highest risk quintile for 10-year non-BC death in BWHS, where the model underestimated survival; results were similar when we examined calibration by age.

**Table 3:** Calibration table for predicting 10-year non-breast cancer death and 5-year breast cancer risk

Predicting 10-year non-breast cancer death												
		NHS development cohort n = 48,102^a^			NHS validation cohort n = 24,088^a^				Black Women’s Health Study n = 15,001			
Risk group^b^	Time (years)	Deaths (%)	Expected survival (using CRR)	Observed survival	Deaths (%)	Expected survival (using CRR)	Observed survival	Expected/observed (E/O)	Deaths (%)	Expected survival (using CRR)	Observed survival	Expected/observed (E/O)
1	5		0.99	0.99		0.99	0.99	1.00		0.99	0.99	1.00
	10	251 (2.6)	0.96	0.98	126 (2.7)	0.96	0.98	0.98	74 (2.2)	0.96	0.98	0.98
2	5		0.98	0.98		0.98	0.98	1.00		0.98	0.98	1.00
	10	553 (5.8)	0.92	0.95	305 (6.3)	0.93	0.94	0.99	220 (5.2)	0.92	0.95	0.97
3	5		0.96	0.97		0.96	0.96	1.00		0.96	0.97	0.99
	10	1133 (11.8)	0.86	0.89	594 (12.2)	0.86	0.89	0.97	284 (8.1)	0.87	0.92	0.95
4	5		0.92	0.93		0.92	0.94	0.98		0.92	0.94	0.98
	10	2320 (24.1)	0.75	0.78	1172 (24.5)	0.75	0.77	0.97	423 (18.0)	0.75	0.83	0.90
5	5		0.73	0.80		0.74	0.80	0.93		0.76	0.84	0.90
	10	5119 (53.2)	0.47	0.49	2593 (53.1)	0.47	0.49	0.96	591 (40.1)	0.44	0.62	0.71

Predicting 5-year invasive breast cancer risk												
		NHS development cohort n = 37,628^a^			NHS validation cohort n = 18,980^a^				Black Women’s Health Study n = 13,247			
Risk group	Time (years)	Invasive breast cancers (%)	Expected survival (using CRR)	Observed survival	Invasive breast cancers (%)	Expected survival (using CRR)	Observed survival	Expected/observed (E/O)	Invasive breast cancers (%)	Expected survival (using CRR)	Observed survival	Expected/observed (E/O)
1	5	80 (1.1)	0.99	0.99	40 (1.1)	0.99	0.99	1.00	40 (1.2)	0.99	0.99	1.00
2	5	122 (1.6)	0.98	0.99	52 (1.3)	0.98	0.99	0.99	40 (1.5)	0.98	0.99	0.99
3	5	112 (1.5)	0.98	0.99	74 (1.9)	0.98	0.98	1.00	39 (1.6)	0.98	0.98	1.00
4	5	150 (2.0)	0.98	0.98	76 (2.1)	0.98	0.98	1.00	50 (2.0)	0.98	0.98	1.00
5	5	248 (3.3)	0.96	0.97	123 (3.3)	0.97	0.97	1.00	54 (2.4)	0.97	0.98	0.99

Calibration of the model in predicting non-BC death was assessed by estimating the ratio of the expected survival (1-CIF for non-BC death from our CRR model) to the observed survival (1-the observed CIF computed using the nonparametric estimation of CIF) at 5 and 10 years within risk quintiles. Similar methods were used to test calibration of the model in predicting BC

CRR = Competing risk regression, CIF = cumulative incidence function, NHS = Nurses’ Health Study, BWHS = Black Women’s Health Study

^a^These analyses include women with complete data. When predicting breast cancer, women with a history of cancer were further excluded

^b^Risk groups were defined by quintiles of the NHS prognostic index for each outcome which was calculated using NHS development cohort regression coefficients

We then applied regression coefficients from the development cohort to the validation cohorts to predict 5-year breast cancer risk. We included all risk factors for non-BC death and breast cancer. That model’s c-index was 0.603 (0.575–0.632) in the NHS validation cohort and 0.556 (0.517–0.595) in the BWHS. We then removed factors that predicted mortality alone (comorbidities, function, and cigarette use); the model’s c-statistic improved to 0.611 (0.582–0.640) in the NHS validation cohort and to 0.566 (0.528–0.604) in BWHS. We then examined model performance considering BMI and alcohol use as continuous rather than categorical variables; model performance improved when using continuous BMI. Finally, we added factors considered in our previous NHS model (months breastfeeding, having a grandmother with breast cancer, and age at menarche), but model performance did not improve. The c-index of our final breast cancer prediction model was 0.612 (0.583–0.641) in the NHS validation cohort and 0.573 (0.536–0.611) in BWHS, as shown in Table 4. Among women aged 55–74, the c-indexes were 0.618 (0.585–0.650) and 0.566 (0.526–0.606) in NHS and BWHS, respectively. Among women ≥ 75, the c-indexes were 0.596 (0.534–0.657) and 0.614 (0.506–0.722) in NHS and BWHS, respectively, in Additional file 1: eTable 9; however, there were only 26 cases among BWHS women ≥ 75. Figure 2 presents 5-year CIFs for breast cancer in each validation cohort. Table 2 and Additional file 1: eTable 7 provide data on the model’s excellent calibration in predicting breast cancer in the validation cohorts. Additional file 1: eTable 8 demonstrates that model performance and HRs are similar using PHR.

**Table 4:** Final model for predicting 5-year breast cancer in the NHS cohorts and BWHS cohort

Predicting 5-year risk of breast cancer	NHS development cohort		NHS validation cohort		BWHS	
Outcome being predicted	HR	*P* value	HR	*P* value	HR	*P* value
	n = 37,628		*n* = 18,980		*n* = 13,247	
N for that outcome	712		365		223	
Factors in our final model						
Age (per year increase)	1.01	0.10	1.01	0.41	1.02	0.15
Highest self-reported Body Mass Index (BMI) in past 10 years: (per kg/m^2^ increase)	1.03	< 0.001	1.02	0.008	1.02	0.01
Average alcohol use per day (highest average use in past 10 years): None	1	–	1	–	1	–
1–4.9 g/day	1.12	0.27	0.99	0.95	0.79	0.17
5–14.9 g/day	1.20	0.09	0.99	0.97	0.95	0.81
15+ g/day	1.29	0.02	1.11	0.52	1.28	0.23
Age at menopause (years), < 45	0.91	0.57	0.64	0.07	1.08	0.74
45–49	1	–	1	–	1	–
50–54	1.21	0.04	1.18	0.20	1.15	0.44
55+	1.32	0.05	1.13	0.53	1.08	0.78
Mammogram in past 2 years | No	1	–	1	–	1	–
Yes	0.91	0.46	0.87	0.40	0.68	0.03
Number of breast biopsies, None	1	–	1	–	1	–
1	1.38	< 0.001	1.51	< 0.001	1.28	0.12
2+	1.31	0.16	1.64	0.04	1.14	0.53
Age at first birth (years) and parity, Nulliparous	1.25	0.23	0.98	0.95	1.10	0.66
< 25, 1–2 children	1	–	1	–	1	–
< 25, 3 + children	1.06	0.62	1.16	0.40	1.02	0.93
25–29, 1–2 children	1.07	0.63	1.12	0.58	1.37	0.15
25–29, 3 + children	1.04	0.76	1.08	0.68	1.60	0.17
30+, 1–2 children	1.37	0.07	2.02	0.002	1.33	0.27
30+, 3 + children	1.06	0.82	0.61	0.31	2.92	0.07
First-degree relatives with history of breast cancer and age at diagnosis, None	1	–	1	–	1	–
1 and age < 50	1.43	0.03	1.30	0.28	1.21	0.56
1 and age 50+	1.30	0.02	1.31	0.08	1.30	0.14
2+ and at least one age < 50	2.19	0.003	3.27	< 0.001	1.60	0.51
2+ and age 50+	2.43	< 0.001	1.53	0.31	0.39	0.35
Postmenopausal hormone use, Never	1	–	1	–	1	–
Current estrogen + progestin user < 5 years	2.28	0.07	1.84	0.38	2.44	0.13
Current estrogen + progestin user 5+ years	2.60	< 0.001	2.87	< 0.001	2.31	0.07
Current estrogen-alone user < 5 years	1.60	0.15	1.42	0.50	1.88	0.14
Current estrogen-alone user 5+ years	1.36	0.02	1.33	0.13	0.98	0.95
Past estrogen + progestin user < 5 years	0.91	0.47	0.82	0.28	0.98	0.91
Past estrogen + progestin user 5+ years	1.17	0.17	1.18	0.30	1.37	0.25
Past estrogen-alone user < 5 years	1.01	0.93	0.78	0.31	1.04	0.84
Past estrogen-alone user 5+ years	0.77	0.06	1.08	0.64	0.70	0.15
C-index (95% CI)	0.610 (0.589–0.631)		0.639 (0.611–0.667)		0.617 (0.582–0.652)	
C-index (95% CI) when using risk factor regression coefficients from the NHS development cohort in the NHS and BWHS validation cohorts	–		0.612 (0.583–0.641)		0.573 (0.536–0.611)	

NHS = Nurses’ Health Study, BWHS = Black Women’s Health Study

### Examples

Table 5 presents risk estimates for four hypothetical women aged 75. Model risk estimates may help identify women at high risk of non-BC death and may provide older women with realistic estimates of their breast cancer risk. Table 6 demonstrates that across cohorts most older women are at low risk of breast cancer and of 10-year non-BC death until age 75 when risk of non-BC death is higher.

**Table 5:** Risk estimates for 5-year breast cancer and 10-year non-BC death, for example, women aged 75

Example cases	Absolute Breast cancer risk	Absolute Breast cancer death risk	Absolute non-Breast cancer death risk	Guidelines recommend^a,b^
Case 1: A 75-year-old woman with BMI = 28, non-smoker, 25 alcoholic drinks per week, average walking pace, no functional limitations, has hypertension, has had a mammogram in the past 2 years, has 1 first-degree relative with breast cancer < 50, had 1 breast biopsy, past user of estrogen + progesterone > 5 years, age 55 at menopause and had 2 children, first birth at 30	3.1% 5-year	0.38% 10-year	11.7% 10-year	Shared decision making
Case 2: A 75-year-old woman with BMI = 23, past smoker, < 2 alcoholic drinks per week, slow walking pace, needs help getting dressed, has a history of cancer, has Parkinson’s, has depression, history of MI, has had a mammogram in the past 2 years, has no family history of breast cancer, age 50 at menopause and had 2 children, first birth at 28	1.7% 5-year	0.27% 10-year	69.8% 10-year	Consider stopping screening
Case 3: A 75-year-old woman with BMI = 22, non-smoker, non-drinker, average walking pace, no functional limitations, has had a mammogram in the past 2 years, age 48 at menopause, had 3 children, first birth at 22	1.3% 5-year	0.15% 10-year	9.8% 10-year	Shared decision making
Case 4: A 75-year-old woman with BMI = 35, former smoker, < 2 alcoholic drinks per week, slow walking pace, limited walking several blocks, has hypertension, diabetes, COPD, history of myocardial infarction, has not had a mammogram in the past 2 years, has 1 first-degree relative with breast cancer < 50, had 2 breast biopsies, past user of estrogen + progesterone > 5 years, age 55 at menopause and had no children	4.3% 5-year	0.44% 10-year	57.8% 10-year	Consider stopping screening

^a^Guidelines: The American Cancer Society and American College of Obstetrics and Gynecology recommend shared decision making around mammography screening for women aged 75 and older and to stop screening when life expectancy is < 10 years. The United States Preventive Services Task Force states the evidence is insufficient to recommend mammography screening to women ≥ 75 but encourages clinicians to be prepared to discuss this service if patients ask [7, 8, 11]

^b^Prior studies have estimated individuals with > 50% 10-year mortality risk to have < 10-year life expectancy since life expectancy is the median survival of a population; therefore, screening is not recommended for these women [38, 39]

**Table 6:** Women at low or high risk of breast cancer and non-BC death in each cohort

	5-year Breast cancer risk (threshold ≥ 3%)a,b	10-year Non-breast cancer death risk (threshold ≥ 50%)a,b	NHS development cohort	NHS validation cohort	BWHS
55–64 years	High	Low	8.1% (939)	7.9% (454)	7.0% (600)
	Low	Low	91.8% (10,586)	92.1% (5317)	93.0% (8031)
	Low	High	0.1% (9)	0.1% (3)	0.1% (5)
	High	High	0.0% (0)	0.0% (0)	0.0% (0)
65–74 years	High	Low	8.3% (1408)	8.2% (694)	5.9% (211)
	Low	Low	90.0% (15,262)	90.1% (7651)	91.6% (3278)
	Low	High	1.5% (257)	1.5% (129)	2.4% (85)
	High	High	0.2% (27)	0.2% (16)	0.2% (6)
75+ years	High	Low	6.6% (603)	6.1% (287)	5.6% (58)
	Low	Low	71.7% (6552)	72.7% (3426)	62.0% (639)
	Low	High	19.8% (1812)	19.5% (920)	30.5% (314)
	High	High	1.9% (173)	1.8% (83)	1.9% (20)

NHS = Nurses’ Health Study, BWHS = Black Women’s Health Study

^a^Guidelines: The American Cancer Society recommends biennial screening for women ≥ 55 at average risk and stopping screening when life expectancy is < 10 years. The American College of Physicians recommends not screening any women ≥ 75 at low or average breast cancer risk or any women with < 10 year life expectancy. The United States Preventive Services Task Force recommends biennial screening for women 50–74 years and states the evidence is insufficient to recommend screening to women ≥ 75 but recommends consideration of women’s breast cancer risk and health [7, 8, 11]

^b^Based on guidelines, we defined breast cancer risk as high when 5-year risk ≥ 3%; otherwise, we defined breast cancer risk as low. We defined mortality risk as high when 10-year mortality risk was ≥ 50%; otherwise, we defined mortality risk as low [36–39]

## Discussion

We developed a novel model to simultaneously predict breast cancer incidence and non-BC death to inform older women’s breast cancer screening decisions. Our model performed well in predicting non-BC death across cohorts. The model slightly over-predicted death in the BWHS after 10 years follow-up in the highest risk group, possibly because data were not available in BWHS on participant mobility and function and these values were highly significant for non-BC death in the NHS cohorts. Our rigorously developed non-BC death prediction model outperformed a model that only included risk factors for death that were selected based on expert opinion. In addition, our model for predicting 5-year breast cancer risk demonstrated excellent calibration but only modest discrimination in predicting breast cancer, similar to the other breast cancer prediction models. Specifically, the Gail and Tyrer-Cuzick breast cancer prediction models have been shown to have c-statistics of < 0.60 in NHS participants ≥ 70 years [20, 46] and breast cancer prediction models developed in case–control studies of breast cancer in Black women have been found to have c-statistics of 0.56 [47] and 0.58 [30] when validated in cohorts of Black women. Our model yielded a similar c-statistic (0.57) in the BWHS.

While mortality indices exist for estimating adults’ 10-year overall mortality risk [37, 48], none specifically predict non-BC death. Although breast cancer death is uncommon and dependent on breast cancer tumor characteristics at diagnosis and treatments received, inclusion of breast cancer death in estimates of overall death when deciding on breast cancer screening may bias decision making. The two most widely used 10-year mortality indices (the Lee and Schonberg indices) were developed from cross-sectional U.S. population surveys and only considered a few diseases [37, 48]. NHS is a longitudinal study which allowed our model to consider numerous diseases and functional measures. While our model includes some risk factors included in these mortality risk models (e.g., age, BMI, smoking history, function, heart failure, emphysema, cancer, and diabetes), our model also considers gait speed, depression, dementia, hypertension, Parkinson’s disease, hip fracture, stroke, and kidney disease. Gait speed is known to be an especially strong predictor of mortality [49]. The inclusion of dementia may be particularly useful since screening decisions for these women may be challenging for caregivers and clinicians [50].

We used competing risk regression to obtain the estimated HRs and CIFs because HRs from competing risk models are directly associated with CIFs and take into account competing risks, while HRs from cause-specific PHR models examine a risk factor’s effect in a hypothetical world with no competing risks. (Competing risks are censored.) Putter et al. have elegantly shown that the HRs from competing risk models are directly associated to those from cause-specific proportional hazard models via the negative logarithm of the reduction factor from the cause-specific model [51]. In our model, hazard ratios were similar regardless of the regression method used, likely because breast cancer death was an uncommon competing risk to non-BC death and non-BC death was an uncommon competing risk to breast cancer incidence. However, our work highlights that the relation between risk factors for breast cancer and non-BC death is complex. Some model risk factors had congruous effects on breast cancer risk and non-BC death, while others had opposing effects. For example, older age was associated with increased risk of both outcomes. Higher BMI was associated with increased breast cancer risk but had a more nuanced effect on non-BC death. Low BMI which may be associated with frailty was highly associated with non-BC death, while BMI > 40 was associated with only a slight increased risk of 10-year non-BC death likely because many of the mediators of high BMI on mortality were included in the model [52]. Greater alcohol consumption was associated with increased breast cancer risk, while drinking < 15 g of alcohol per day (<1 drink) was associated with lower non-BC death risk.

In addition, while the hazard ratios associated with many of the risk factors for non-BC death differed significantly between the predominantly white women in NHS and the Black women in BWHS, qualitative trends were similar. When predicting breast cancer, only the HRs for increasing BMI and prior mammography differed significantly between the cohorts; however, increasing BMI was associated with increasing breast cancer risk in both cohorts and having a mammogram in the past 2 years was associated with women being less likely to be diagnosed with breast cancer in 5 years. Simulation modelers have also found that it is necessary to consider women’s prior use of mammography in estimating breast cancer incidence [53]. While we initially planned to include all the non-BC death risk factors in our competing risk breast cancer prediction model, we found that doing so led to our model performing less well likely because the model was overfit. Instead, the model performed better in validation when only including breast cancer risk factors; importantly, some breast cancer risk factors (e.g., age, alcohol use, age at menopause) also predicted non-BC death.

While our rigorously developed model predicts breast cancer and non-breast cancer death, it also has limitations. Since actual mammographic density was not available for most participants, we considered predicted mammographic density. However, we did not find an association with breast cancer risk likely because our model already included several factors associated with breast density (e.g., BMI) and because the prevalence of high mammographic density decreases with age as does its effect on breast cancer risk [54–56]. Furthermore, inclusion of polygenic risk scores (PRS) in breast cancer prediction models has been shown to increase model discrimination slightly; however, the association of PRS with breast cancer risk declines with age and few women have obtained this information [57–59]. If PRS data become more available in practice, we would test adding PRS to the model in the future. In addition, model performance needs to be tested in older Asian and Hispanic women before being used in these populations.

## Conclusions

We formally modeled prediction of breast cancer and non-BC death in a competing risk model we are developing for clinical use. As demonstrated with the examples in Table 4, model risk estimates may be helpful in identifying women at high risk of non-BC death who are unlikely to benefit from screening and in providing older women with realistic estimates of their breast cancer risk. Before making our model available online, we plan to formally compare performance of our model to existing breast cancer prediction models such as the BCRAT and Tyrer-Cuzick [60] and to further examine its performance in other diverse cohorts such as Women’s Health Initiative and Multiethnic Cohort [61, 62].

## Supplementary Information

**Additional file 1**. Supplementary materials.

## Data Availability

Investigators who wish to use data collected in the NHS are encouraged to visit http://www.nurseshealthstudy.org/researchers.

## Abbreviations

AIC: Akaike information criterion
BC: Breast cancer
BMI: Body mass index
BWHS: Black Women’s Health Study
CIF: Cumulative incidence function
CRR: Competing risk regression
HR: Hazard ratio
MHT: Menopausal hormone therapy
NHS: Nurses’ Health Study
SES: Socioeconomic status
USA: United States of America
