# Supplementary material for: A model for predicting both breast cancer risk and non-breast cancer death among women > 55 years old
Source: Breast Cancer Res. 2023 Jan 24;25:8. doi: 10.1186/s13058-023-01605-8 (PMC9872276; doi:10.1186/s13058-023-01605-8)
Supplement: Supplementary file 1 — Additional file 1. Supplementary materials. [file 13058_2023_1605_MOESM1_ESM.docx]

Supplementary Information Table of Contents:

1. eTable 1: Baseline characteristics among participants in the NHS cohorts and in BWHS by age …page 2.
2. eTable 2: List of NHS variables considered in predicting non-breast cancer death and reasons variables were removed…………………………………………………………………………………………..….page 5.
3. eTable 3: Age adjusted c-statistic in predicting 10-year non-breast cancer death in the NHS development cohort ………………………………………………………………….………….............................page 7.
4. eTable 4: Using multiple imputation in predicting 10-year non-breast cancer death in the NHS development cohort…………………………………………………………………………………………….…..page 8.
5. eTable 5: Cause-Specific Proportional Hazards Regression Model in predicting 10-year non-BC death in the NHS Development Cohort.…………………………………………………………………………..page 10
6. eTable 6: Final competing risk regression model for predicting 10-year non-breast cancer death by age across cohorts………………………………………………………..…………………………...…..page 12.
7. eTable 7: Calibration table for predicting 10-year non-breast cancer death and 5-year breast cancer risk by age…………………………………………………………………………………………………….page 14
8. eTable 8: Cause-Specific Proportional Hazards Regression Model in predicting 5-year breast cancer risk in NHS development cohort…..………………………………………………………………………..page 16.
9. eTable 9: Final competing risk regression model for predicting 5-year breast cancer risk by age across cohorts……………………………………………….…………………………………………...…..page 18
10. eFigure 1: Sample population………………………………………………………………………...page 20
11. Appendix A: Description of the Nurses’ Health Study and Black Women’s Health Study………….page 21
12. Appendix B: Data Dictionary: Nurses’ Health Study and Black Women’s Health Study…………...page 22
13. Appendix C. More Detailed Description of Methods for validating the CRR model and multiple imputation ………………………………………………………...........................................................................page 35.

eTable 1: Baseline characteristics among participants in the NHS development and validation cohorts and in BWHS by age.^a^

|  | NHS Development Cohort | | NHS Validation Cohort | | BWHS Cohort | |
| --- | --- | --- | --- | --- | --- | --- |
|  | 55-74 | 75+ | 55-74 | 75+ | 55-74 | 75+ |
| N | 39,897 | 15,656 | 19,787 | 7,990 | 15,946 | 1,434 |
| Factors in our final model |  |  |  |  |  |  |
| Age, mean (SD) | 66.6 (4.8) | 79.0 (2.4) | 66.6 (4.8) | 79.0 (2.4) | 62.3 (5.2) | 78.6 (2.5) |
| 55-59 years, % | 9.9 | - | 9.4 | - | 39.8 | - |
| 60-64 years, % | 29.8 | - | 30.2 | - | 30.8 | - |
| 65-69 years, % | 31.2 | - | 31.3 | - | 18.6 | - |
| 70-74 years, % | 29.1 | - | 29.1 | - | 10.8 | - |
| 75-79 years, % | - | 64.2 | - | 65.2 | - | 70.4 |
| 80+ years, % | - | 35.8 | - | 34.8 | - | 29.6 |
| Highest self-reported Body Mass Index (BMI) in past 10 years kg/m^2^ , mean (SD) | 28.5 (5.9) | 27.1 (5.1) | 28.5 (6.0) | 27.0 (5.1) | 32.3 (7.1) | 30.5 (5.9) |
| Highest self-reported BMI in past 10 years |  |  |  |  |  |  |
| <20 kg/m^2^, % | 2.2 | 4.0 | 2.2 | 4.0 | 0.5 | 0.6 |
| 20-22.4 kg/m^2^, % | 10.2 | 12.8 | 10.4 | 13.2 | 3.0 | 3.3 |
| 22.5-24.9 kg/m^2^, % | 18.2 | 21.3 | 18.7 | 21.3 | 8.2 | 11.9 |
| 25-29.9 kg/m^2^, % | 36.0 | 37.9 | 35.8 | 37.6 | 31.1 | 37.8 |
| 30-34.9 kg/m^2^, % | 20.4 | 17.1 | 19.6 | 17.0 | 28.4 | 26.6 |
| 35-39.9 kg/m^2^, % | 8.0 | 4.8 | 8.3 | 4.7 | 14.8 | 10.7 |
| 40+ kg/m^2^, % | 4.9 | 2.0 | 4.9 | 2.1 | 13.1 | 7.6 |
| Unknown, % | 0.1 | 0.2 | 0.1 | 0.2 | 1.0 | 1.5 |
| Average alcohol use per day (highest average use in past 10 years) ^b^ |  |  |  |  |  |  |
| None, % | 33.4 | 46.0 | 33.4 | 46.0 | 49.6 | 58.8 |
| 1-4.9 gram/day, % | 24.5 | 17.4 | 24.8 | 17.5 | 26.4 | 22.0 |
| 5-14.9 gram/day, % | 18.2 | 15.7 | 17.6 | 15.8 | 14.0 | 10.9 |
| 15+ gram/day, % | 13.5 | 12.1 | 14.0 | 11.9 | 10.0 | 8.4 |
| Unknown, % | 10.4 | 8.8 | 10.2 | 8.9 | - | - |
| Cigarette use |  |  |  |  |  |  |
| Never | 43.5 | 47.6 | 43.8 | 47.2 | 53.9 | 48.1 |
| Current | 8.8 | 5.2 | 9.2 | 4.8 | 10.6 | 5.2 |
| Past | 47.6 | 47.0 | 46.9 | 47.8 | 35.5 | 46.8 |
| Unknown | 0.2 | 0.3 | 0.2 | 0.3 | - | - |
| Limited from walking several blocks |  |  |  |  |  |  |
| Not at all | 68.7 | 44.3 | 68.6 | 44.1 | - | - |
| A little or a lot | 25.7 | 51.0 | 25.9 | 50.8 | - | - |
| Unknown, % | 5.6 | 4.7 | 5.5 | 5.1 | - | - |
| Limited in bathing or dressing oneself |  |  |  |  |  |  |
| Not at all | 89.7 | 84.4 | 89.5 | 83.9 | - | - |
| A little or a lot | 4.7 | 11.0 | 5.1 | 11.2 | - | - |
| Unknown, % | 5.6 | 4.6 | 5.4 | 5.0 | - | - |
| Usual walking pace outdoors |  |  |  |  |  |  |
| Unable to walk^c^ | 1.7 | 4.8 | 1.8 | 5.2 | - | - |
| Slow or average (less than 3mph) | 69.6 | 82.2 | 69.3 | 80.7 | 61.1 | 71.4 |
| Brisk/very brisk (>3mph) | 24.0 | 8.8 | 24.2 | 9.7 | 28.6 | 14.6 |
| Unknown, % | 4.8 | 4.2 | 4.7 | 4.3 | 10.3 | 14.0 |
| High Blood pressure^d^ | 56.1 | 71.1 | 56.0 | 70.8 | 67.7 | 80.1 |
| Depression | 21.1 | 18.5 | 21.3 | 18.5 | 22.8 | 15.8 |
| Hip Fracture | 1.2 | 4.3 | 1.2 | 4.7 | 0.7 | 1.8 |
| Parkinson’s Disease | 0.4 | 1.3 | 0.4 | 1.2 | 0.1 | 0.7 |
| Myocardial Infarction | 4.1 | 9.1 | 4.2 | 9.6 | 3.7 | 10.6 |
| Congestive heart failure | 2.0 | 6.5 | 2.0 | 6.8 | 2.9 | 6.9 |
| Stroke/Transient Ischemic Attack | 4.8 | 14.3 | 5.0 | 13.8 | 3.7 | 8.9 |
| Emphysema/Asthma | 18.0 | 20.1 | 18.2 | 19.4 | 18.3 | 17.7 |
| Diabetes | 11.3 | 14.5 | 10.8 | 14.2 | 23.5 | 31.5 |
| Dementia | 0.5 | 3.1 | 0.5 | 3.0 | 0.1 | 1.2 |
| Kidney Disease | 0.5 | 0.7 | 0.5 | 0.7 | 1.8 | 2.9 |
| Cancer^e^ | 10.6 | 16.7 | 10.4 | 16.5 | 5.3 | 9.8 |
| Age at menopause (years) |  |  |  |  |  |  |
| <45, % | 11.2 | 9.6 | 10.9 | 9.2 | 18.8 | 24.2 |
| 45-49, % | 23.1 | 24.0 | 23.7 | 24.4 | 20.4 | 17.5 |
| 50-54, % | 54.9 | 59.3 | 55.0 | 59.3 | 27.7 | 24.6 |
| 55+, % | 9.4 | 7.0 | 9.0 | 7.0 | 7.8 | 13.5 |
| Hysterectomy, age at menopause unknown, % ^f^ | - | - | - | - | 22.4 | 20.0 |
| Unknown, % | 1.3 | 0.2 | 1.4 | 0.1 | 2.9 | 0.1 |
| Mammogram in past 2 years ^g^ |  |  |  |  |  |  |
| No | 10.1 | 17.2 | 10.2 | 17.2 | 13.5 | 19.5 |
| Yes | 81.4 | 74.4 | 81.6 | 74.5 | 86.5 | 80.5 |
| Unknown | 8.5 | 8.4 | 8.3 | 8.4 | - | - |
| Number of breast biopsies |  |  |  |  |  |  |
| 0, % | 72.3 | 75.6 | 72.2 | 75.9 | 67.2 | 64.9 |
| 1, % | 24.1 | 21.9 | 24.3 | 21.8 | 21.4 | 20.5 |
| 2+, % | 3.6 | 2.6 | 3.6 | 2.3 | 11.5 | 14.6 |
| Postmenopausal hormone use |  |  |  |  |  |  |
| Never, % | 21.9 | 24.0 | 22.3 | 24.4 | 40.5 | 42.7 |
| Current estrogen plus progestin user <5 years, % | 0.6 | 0.1 | 0.6 | 0.1 | 0.7 | 0.0 |
| Current estrogen plus progestin user 5+ years, % | 3.4 | 0.8 | 3.4 | 1.0 | 1.2 | 0.5 |
| Current estrogen-alone user <5 years, % | 1.1 | 0.4 | 1.0 | 0.4 | 1.8 | 0.1 |
| Current estrogen-alone user 5+ years, % | 10.3 | 7.0 | 10.0 | 6.9 | 6.5 | 4.4 |
| Past estrogen plus progestin user <5 years, % | 16.8 | 10.1 | 16.6 | 10.6 | 13.1 | 8.9 |
| Past estrogen plus progestin user 5+ years, % | 18.8 | 8.1 | 19.1 | 8.6 | 5.5 | 6.6 |
| Past estrogen-alone user <5 years, % | 5.8 | 10.4 | 5.8 | 10.6 | 12.1 | 12.5 |
| Past estrogen-alone user 5+ years, % | 12.8 | 16.2 | 12.9 | 15.8 | 13.3 | 19.2 |
| Unknown, % | 8.5 | 22.9 | 8.3 | 21.8 | 5.3 | 5.2 |
| Age at first birth (years) and parity^h^ |  |  |  |  |  |  |
| Nulliparous | 4.9 | 6.2 | 5.1 | 6.1 | 16.5 | 10.5 |
| <25, 1-2 children | 16.3 | 8.9 | 16.1 | 8.5 | 33.3 | 20.2 |
| <25, 3+ children | 37.9 | 28.4 | 37.1 | 29.4 | 26.5 | 42.3 |
| 25-29, 1-2 children | 15.7 | 12.6 | 16.2 | 13.2 | 12.1 | 10.0 |
| 25-29, 3+ children | 16.8 | 28.5 | 17.2 | 27.9 | 2.6 | 6.6 |
| 30+, 1-2 children | 5.0 | 8.0 | 5.0 | 7.8 | 7.1 | 7.0 |
| 30+, 3+ children | 1.8 | 5.4 | 1.8 | 5.4 | 0.5 | 1.1 |
| Unknown | 1.7 | 1.9 | 1.6 | 1.7 | 1.6 | 2.4 |
| Number of first-degree relatives with history of breast cancer and age at diagnosis^i^ |  |  |  |  |  |  |
| None, % | 83.2 | 79.3 | 83.1 | 79.0 | 80.1 | 80.4 |
| 1 and age <50, % | 4.1 | 4.6 | 3.9 | 4.6 | 3.7 | 5.2 |
| 1 and age 50+, % | 11.1 | 12.8 | 11.3 | 13.1 | 14.4 | 12.0 |
| 2+ and at least one age <50 , % | 0.8 | 1.5 | 1.0 | 1.6 | 0.6 | 0.7 |
| 2+ and age 50+, % | 0.8 | 1.8 | 0.8 | 1.7 | 1.3 | 1.7 |
| Outcomes^j^ |  |  |  |  |  |  |
| Breast cancer in 5-year follow-up, % | 1.8 | 1.6 | 1.7 | 1.7 | 1.7 | 2.2 |
| Breast cancer in 10-year follow-up, % | 3.4 | 2.4 | 3.4 | 2.8 | 3.1 | 3.1 |
| Breast cancer death in 5-year follow-up, % | 0.1 | 0.2 | 0.04 | 0.1 | 0.2 | 0.1 |
| Breast cancer death in 10-year follow-up, % | 0.2 | 0.4 | 0.2 | 0.4 | 0.4 | 0.8 |
| Non-breast cancer death 5-year follow-up, % | 4.1 | 16.7 | 4.7 | 16.3 | 3.4 | 16.0 |
| Non-breast cancer death 10-year follow-up, % | 11.5 | 42.1 | 12.0 | 42.0 | 8.7 | 37.5 |
| Not included in model |  |  |  |  |  |  |
| Race/ethnicity |  |  |  |  |  |  |
| Non-Hispanic White,% | 96.2 | 96.3 | 96.2 | 96.7 | 0.0 | 0.0 |
| Non-Hispanic Black,% | 1.8 | 1.7 | 1.7 | 1.5 | 99.1 | 99.3 |
| Hispanic,% | 1.0 | 0.9 | 0.9 | 1.0 | 0.9 | 0.7 |
| Asian, Pacific Islander % | 0.8 | 0.9 | 1.0 | 0.7 | 0.0 | 0.0 |
| Native American,% | 0.3 | 0.2 | 0.2 | 0.2 | 0.0 | 0.0 |
| Education (years)^k^ |  |  |  |  |  |  |
| <12 | - | - | - | - | 2.4 | 6.2 |
| 12 | - | - | - | - | 15.9 | 21.5 |
| 13-15 | - | - | - | - | 28.3 | 22.3 |
| 16 | 90.7 | 94.0 | 90.6 | 94.2 | 19.8 | 16.7 |
| 17+ | 9.3 | 6.0 | 9.4 | 5.8 | 33.6 | 33.3 |
| Unknown | - | - | - | - | 0.1 | 0.1 |
| Predicted Breast density^l^ | n=25,694 | n=9,379 | n=12,699 | n=4,783 |  |  |
| Higher than median (>24.7% dense) | 54.9 | 36.5 | 55.8 | 34.8 | - | - |
| Lower than median (<24.7% dense) | 45.1 | 63.5 | 44.2 | 65.2 | - | - |

a. NHS included participants that completed the 2004 questionnaire. BWHS included participants that completed the 2009 questionnaire.

b. A standard drink is any drink that contains about 14 grams of pure alcohol (12 oz. of beer, 5 oz. of wine or 1.5 oz. of liquor)

c. BWHS does not ask about participants’ ability to walk.

d. Health conditions were self-reported.

e. Excluded breast and non-melanomatous skin cancers.

f. Participants with hysterectomy and age at menopause unknown in BWHS were placed in the 45-49 years category for all analyses; Sensitivity analyses were performed showing no significant difference when placed in any other age category.

g. Participants missing data on mammography use in NHS had completed a short version of the 2004 questionnaire.

h. Participants who were parous with an unknown number of children were categorized as having 1-2 children.

i. Relatives with an unknown age at diagnosis were categorized as having been diagnosed at 50+ years of age.

j. Participants were followed for 10 years after start of follow-up.

k. Participants in NHS who had a master’s or doctorate degree were placed in the 17+ years category. All others were placed in the 16 years category, as registered nurses, some of whom have a bachelor’s degree (22.8%).

l. Predicted breast density was defined using a 9-item validated index. Prevalence is reported for those with complete data.^18^

eTable 2: List of NHS variables considered in predicting non-breast cancer death and reasons variables were removed.^a^

| **Variable** | **In Model?** | **Reason Removed** |
| --- | --- | --- |
| Age | Yes |  |
| Age of death of biological mother/father | No | 22.8% missing data |
| Age at menopause | Yes |  |
| ***Health Behaviors (4)*** |  |  |
| BMI | Yes |  |
| Alcohol use | Yes |  |
| Cigarette use | Yes |  |
| Use of sugary drinks | No | 7.3% missing and 3 questions needed to be answered to define |
| ***Comorbidities (32)*** |  |  |
| Myocardial infarction | Yes |  |
| Stroke/Transient ischemic attack | Yes |  |
| Congestive heart failure | Yes |  |
| Angina | No | Not associated with non-breast cancer death in univariate analyses |
| Diabetes | Yes |  |
| High blood pressure | Yes |  |
| Peripheral vascular disease | No | Eliminated after best subsets regression |
| Hip fracture | Yes |  |
| Wrist fracture | No | Difficult for women to self-report if non-traumatic |
| Vertebral fracture | No | Difficult to self-report |
| Chronic kidney disease | Yes |  |
| Dementia/Alzheimer's | Yes |  |
| GI bleed requiring hospitalization | No | Eliminated after best subsets regression |
| Parkinson's disease | Yes |  |
| Multiple Sclerosis | No | Eliminated after best subsets regression |
| Seizures/epilepsy | No | Eliminated after best subsets regression |
| Connective tissue disease | No | Eliminated after best subsets regression also there is inaccurate self-reporting of rheumatoid arthritis |
| Emphysema/asthma | Yes |  |
| Osteoarthritis | No | Difficult to self-report, other functional variables are easier to report |
| Osteoporosis | No | Difficult to self-report, women confuse osteoporosis with osteoarthritis |
| Hypothyroidism | No | Eliminated after best subsets regression |
| Hyperthyroidism | No | Eliminated after best subsets regression |
| Inflammatory Bowel Disease | No | Eliminated after best subsets regression |
| Amyotrophic lateral sclerosis (ALS) | No | Prevalence <0.1% |
| Pneumonia | No | Not significant in validation cohort |
| Peptic Ulcer | No | Eliminated after best subsets regression |
| AIDS | No | Prevalence <0.1% |
| Cirrhosis | No | Prevalence <0.1% |
| Mild liver disease | No | Eliminated after best subsets regression |
| Gout | No | Did not improve the c-statistic of the final model |
| Cancer | Yes |  |
| Depression/use of anti-depressants | Yes |  |
| ***Measures of Physical Function (16)*** |  |  |
| Walking pace | Yes |  |
| METs per week | No | Too many questions needed to obtain data |
| Flights of stairs climbed daily | No | Difficult to accurately self-report and to compute quickly |
| Limited walking several blocks | Yes |  |
| Limited in vigorous activity | No | Not significant in final model |
| Limited in moderate activity (moving a table, vacuuming) | No | Highly correlated with limited walking (r=0.57) |
| Limited in lifting/carrying groceries | No | Highly correlated with limited walking (r=0.64) |
| Limited climbing stairs | No | Highly correlated with limited walking (r=0.62) |
| Limited bending/kneeling/stooping | No | Highly correlated with limited walking (r=0.55) |
| Limited bathing/dressing | Yes |  |
| Physical health cutting down on work time/difficulty working | No | 10.0% missing data |
| Amount of bodily pain | No | 9.9% missing data |
| 1+ fall in past year | No | Eliminated after best subsets regression |
| Use of cane/walker | No | 15.2% missing data |
| Difficulty with balance | No | Due to being correlated with difficulty with walking at 0.36 and to keep the model parsimonious |
| Use of statins | No | May be given for primary or secondary prevention and the hazard ratio combines these two different populations |
| ***Psychosocial (5)*** |  |  |
| Amount of emotional support | No | 10.5% missing data |
| How many people provide emotional support | No | 10.4% missing data |
| Physical health/emotional problems interfering with social activities |  | 10.0% missing data |
| Anxiety | No | 10.7% missing data |
| Perceived health | No | 15.8% missing data, last assessed in 2000 meaning that many in fair/poor health had passed before the 2004 questionnaire |

1. Best subsets regression led to 961 total models. Of these, 281 had a c-statistic of 0.789 (the highest c-statistic). Within this group the AIC varied by <0.02%. We considered the variables that made it into at least 97% of the top 281 models. The next variables only made it into fewer than 84% of the top models, they were rare (e.g, multiple sclerosis) and/or difficult to accurately self-report (e.g., connective tissue disease) and their removal did not affect model performance.

eTable 3: Age adjusted c-statistic in predicting 10-year non-breast cancer death in the NHS development cohort.

| Predicting 10-year non-breast cancer death (n=48,102) | | | | | |
| --- | --- | --- | --- | --- | --- |
| Age | N | Events | c-statistic | Standard Error | Age adjusted c-statistic |
| 55-74 | 34292 | 3778 | 0.7652 | 0.0038 |  |
| 75+ | 13810 | 5598 | 0.6986 | 0.0035 |  |
|  |  |  |  |  | 0.7292 |

eTable 4: Using multiple imputation in predicting 10-year non-breast cancer death in the NHS development cohort.

|  | Base model missing as a category  (n=55,553) | | | Multiple Imputation  (n=55,553) | | | Complete Case Analysis  (n=48,102) | | |
| --- | --- | --- | --- | --- | --- | --- | --- | --- | --- |
|  | P value | Hazard Ratio | Prevalence | P value | Hazard Ratio | Prevalence | P value | Hazard Ratio | Prevalence |
| N for that outcome | 11165 |  |  | 11165 |  |  | 9388 |  |  |
| Factors in our final model |  |  |  |  |  |  |  |  |  |
| Age | <0.001 | 1.11 (1.11-1.12) |  | <0.001 | 1.11 (1.11-1.11) |  | <0.001 | 1.11 (1.11-1.12) |  |
| Highest self-reported Body Mass Index (BMI) in past 10 years: <20 kg/m^2^ | <0.001 | 1.56 (1.41-1.72) | 2.7 | <0.001 | 1.58 (1.43-1.74) | 2.7 | <0.001 | 1.63 (1.45-1.82) | 2.7 |
| 20-22.4 kg/m^2^ | <0.001 | 1.16 (1.08-1.24) | 10.9 | <0.001 | 1.16 (1.08-1.24) | 11.0 | <0.001 | 1.15 (1.07-1.25) | 11.2 |
| 22.5-24.9 kg/m^2^ | - | 1 | 19.1 | - | 1 | 19.1 | - | 1 | 19.6 |
| 25-29.9 kg/m^2^ | <0.001 | 0.90 (0.86-0.95) | 36.5 | <0.001 | 0.91 (0.86-0.96) | 36.6 | 0.003 | 0.91 (0.86-0.97) | 36.9 |
| 30-34.9 kg/m^2^ | <0.001 | 0.87 (0.82-0.93) | 19.5 | <0.001 | 0.88 (0.83-0.94) | 19.5 | <0.001 | 0.88 (0.82-0.94) | 19.1 |
| 35-39.9 kg/m^2^ | 0.008 | 0.88 (0.81-0.96) | 7.1 | 0.008 | 0.89 (0.82-0.97) | 7.1 | 0.07 | 0.91(0.83-1.01) | 6.9 |
| 40+ kg/m^2^ | 0.06 | 1.11 (1.00-1.23) | 4.1 | 0.06 | 1.10 (0.99-1.22) | 4.1 | 0.10 | 1.10 (0.98-1.24) | 3.8 |
| Missing | 0.28 | 1.29 (0.81-1.92) | 0.1 | - | - | - | - | - | - |
| Average alcohol use per day (highest average use in past 10 years) : None | - | 1 | 37.0 | - | 1 | 41.2 | - | 1 | 40.7 |
| 1-4.9 gram/day | <0.001 | 0.86 (0.82-0.91) | 22.5 | <0.001 | 0.88 (0.84-0.92) | 24.9 | <0.001 | 0.86 (0.82-0.91) | 25.0 |
| 5-14.9 gram/day | 0.003 | 0.91 (0.86-0.97) | 17.5 | 0.001 | 0.92 (0.87-0.97) | 19.4 | 0.004 | 0.91 (0.86-0.97) | 19.5 |
| 15+ gram/day | 0.36 | 1.03 (0.97-1.10) | 13.1 | 0.65 | 1.02 (0.96-1.08) | 14.5 | 0.24 | 1.04 (0.97-1.11) | 14.8 |
| Missing | 0.89 | 1.01 (0.92-1.10) | 10.0 | - | - | - | - | - | - |
| Cigarette use: Never | - | 1 | 44.7 | - | 1 | 44.7 | - | 1 | 44.8 |
| Current | <0.001 | 2.46 (2.31-2.63) | 7.7 | <0.001 | 2.49 (2.33-2.65) | 7.8 | <0.001 | 2.46 (2.28-2.65) | 7.3 |
| Past | <0.001 | 1.36 (1.30-1.41) | 47.4 | <0.001 | 1.35 (1.30-1.41) | 47.5 | <0.001 | 1.35 (1.29-1.42) | 47.9 |
| Missing | 0.87 | 1.04 (0.66-1.53) | 0.2 | - | - | - | - | - |  |
| Limited from walking several blocks: Not at all | - | 1 | 61.8 | - | 1 | 65.2 | - | 1 | 64.7 |
| A little/a lot | <0.001 | 1.53 (1.46-1.60) | 32.8 | <0.001 | 1.51 (1.46-1.59) | 34.8 | <0.001 | 1.58 (1.50-1.66) | 35.3 |
| Missing | 0.005 | 1.87 (1.25-2.68) | 5.4 | - | - | - | - | - | - |
| Limited in bathing/dressing oneself: Not at all | - | 1 | 88.2 | - | 1 | 93.2 | - | 1 | 93.2 |
| A little or a lot | <0.001 | 1.43 (1.35-1.51) | 6.5 | <0.001 | 1.43 (1.35-1.51) | 6.8 | <0.001 | 1.47 (1.38-1.57) | 6.8 |
| Missing | 0.03 | 0.62 (0.43-0.92) | 5.3 | - | - | - | - | - |  |
| Walking pace: Unable to walk | <0.001 | 1.43 (1.32-1.54) | 2.6 | <0.001 | 1.39 (1.29-1.50) | 2.7 | <0.001 | 1.42 (1.30-1.55) | 2.8 |
| Slow or average (<3mph) | - | 1 | 73.1 | - | 1 | 76.6 | - | 1 | 76.6 |
| Brisk/very brisk (>3mph) | <0.001 | 0.69 (0.64-0.75) | 19.7 | <0.001 | 0.72 (0.67-0.77) | 20.6 | <0.001 | 0.69 (0.64-0.74) | 20.6 |
| Missing | 0.44 | 0.95 (0.85-1.07) | 4.6 | - | - | - |  |  | - |
| High blood pressure | <0.001 | 1.15 (1.10-1.20) | 60.3 | <0.001 | 1.16 (1.11-1.21) | 60.3 | <0.001 | 1.15 (1.09-1.21) | 59.9 |
| Depression | <0.001 | 1.17 (1.12-1.22) | 20.4 | <0.001 | 1.16 (1.11-1.22) | 20.4 | <0.001 | 1.17 (1.11-1.23) | 20.4 |
| Hip fracture | <0.001 | 1.39 (1.27-1.51) | 2.1 | <0.001 | 1.38 (1.26-1.50) | 2.1 | <0.001 | 1.31 (1.18-1.45) | 2.0 |
| Parkinson’s disease | <0.001 | 2.39 (2.09-2.72) | 0.7 | <0.001 | 2.42 (2.12-2.74) | 0.7 | <0.001 | 2.35 (1.98-2.79) | 0.6 |
| Myocardial infarction | <0.001 | 1.25 (1.18-1.33) | 5.5 | <0.001 | 1.26 (1.18-1.34) | 5.5 | <0.001 | 1.27 (1.18-1.37) | 5.4 |
| Congestive heart failure | <0.001 | 1.75 (1.64-1.88) | 3.3 | <0.001 | 1.76 (1.64-1.88) | 3.3 | <0.001 | 1.77 (1.62-1.92) | 3.1 |
| Stroke/Transient Ischemic Attack | <0.001 | 1.23 (1.17-1.30) | 7.5 | <0.001 | 1.24 (1.18-1.31) | 7.5 | <0.001 | 1.19 (1.12-1.27) | 7.2 |
| Emphysema/Asthma | <0.001 | 1.27 (1.22-1.33) | 18.6 | <0.001 | 1.28 (1.22-1.33) | 18.6 | <0.001 | 1.29 (1.22-1.35) | 18.5 |
| Diabetes | <0.001 | 1.38 (1.31-1.45) | 12.2 | <0.001 | 1.40 (1.33-1.47) | 12.3 | <0.001 | 1.36 (1.29-1.45) | 11.6 |
| Dementia | <0.001 | 3.14 (2.87-3.44) | 1.2 | <0.001 | 3.31 (3.02-3.62) | 1.2 | <0.001 | 3.07 (2.69-3.50) | 1.0 |
| Kidney disease | <0.001 | 1.51 (1.26-1.79) | 0.5 | <0.001 | 1.56 (1.32-1.88) | 0.6 | 0.07 | 1.28 (0.98-1.66) | 0.5 |
| Cancer* | <0.001 | 1.41 (1.34-1.48) | 12.3 | <0.001 | 1.42 (1.35-1.49) | 12.3 | <0.001 | 1.45 (1.37-1.53) | 12.4 |
| Age at menopause (years), <45 | 0.67 | 1.02 (0.95-1.08) | 10.8 | 0.61 | 1.02 (0.95-1.08) | 10.9 | 0.74 | 1.01 (0.94-1.09) | 10.9 |
| 45-49 | - | 1 | 23.4 | - | 1 | 23.6 | - | 1 | 23.7 |
| 50-54 | 0.01 | 0.94 (0.90-0.99) | 56.1 | 0.01 | 0.94 (0.90-0.99) | 56.7 | 0.004 | 0.93 (0.88-0.98) | 56.6 |
| 55+ | 0.006 | 0.89 (0.82-0.97) | 8.8 | 0.007 | 0.89 (0.83-0.97) | 8.8 | 0.002 | 0.87 (0.79-0.95) | 8.8 |
| Missing | 0.98 | 1.00 (0.74-1.32) | 1.0 | - | - | - | - | - | - |
| Mammogram in past 2 years\|\| No | - | 1 | 12.1 | - | 1 | 13.3 | - | 1 | 12.9 |
| Yes | <0.001 | 0.75 (0.72-0.79) | 79.4 | <0.001 | 0.75 (0.72-0.79) | 86.7 | <0.001 | 0.75 (0.71-0.79) | 87.1 |
| Missing | 0.001 | 1.21 (1.10-1.35) | 8.5 | - | - | - | - | - | - |
| C-index (95% CI) |  | 0.796 (0.792-0.800) |  | 0.795 (0.795-0.796) |  |  |  | 0.795 (0.791-0.800) |  |
| AIC |  | 227974.5 |  | 228008.5 |  |  |  | 188839.2 |  |

eTable 5: Cause-Specific Proportional Hazards Regression Model in predicting 10-year non-BC death in the NHS Development Cohort.^a,b^

| Predicting 10-year non-breast cancer death | Predicting non-breast cancer death | | | | |
| --- | --- | --- | --- | --- | --- |
|  | Competing Risk Regression (CRR) | | Cause-specific Proportional Hazards regression | |  |
|  | HR  n=48,102 | P value | HR  n=48,102 | P value | P value for difference in risk factor regression coefficients from CRR model |
| N for outcome | 9,376 |  | 9,376 |  |  |
| Factors in our final model |  |  |  |  |  |
| Age (per year of age increase) | 1.11 | <0.001 | 1.11 | <0.001 | 0.93 |
| Highest self-reported Body Mass Index (BMI) in past 10 years: <20 kg/m^2^ | 1.63 | <0.001 | 1.62 | <0.001 | 0.99 |
| 20-22.4 kg/m^2^ | 1.15 | <0.001 | 1.15 | <0.001 | 0.98 |
| 22.5-24.9 kg/m^2^ | 1 | - | 1 | - | - |
| 25-29.9 kg/m^2^ | 0.91 | 0.003 | 0.91 | 0.003 | 0.99 |
| 30-34.9 kg/m^2^ | 0.88 | <0.001 | 0.88 | <0.001 | 0.96 |
| 35-39.9 kg/m^2^ | 0.91 | 0.07 | 0.91 | 0.06 | 0.99 |
| 40+ kg/m^2^ | 1.10 | 0.1 | 1.10 | 0.08 | 0.97 |
| Average alcohol use per day (highest average use in past 10 years) :¶ None | 1 | - | 1 | - | - |
| 1-4.9 gram/day | 0.86 | <0.001 | 0.86 | <0.001 | 0.99 |
| 5-14.9 gram/day | 0.91 | 0.004 | 0.91 | 0.003 | 0.99 |
| 15+ gram/day | 1.04 | 0.24 | 1.04 | 0.19 | 0.93 |
| Cigarette use: Never | 1 | - | 1 | - | - |
| Current | 2.46 | <0.001 | 2.47 | <0.001 | 0.94 |
| Past | 1.35 | <0.001 | 1.35 | <0.001 | 0.99 |
| Limited from walking several blocks: Not at all | 1 | - | 1 | - | - |
| A little/a lot | 1.58 | <0.001 | 1.58 | <0.001 | 0.99 |
| Limited in bathing/dressing oneself: Not at all | 1 | - | 1 | - | - |
| A little or a lot | 1.47 | <0.001 | 1.47 | <0.001 | 0.99 |
| Walking pace: Unable to walk | 1.42 | <0.001 | 1.42 | <0.001 | 0.93 |
| Slow or average (<3mph) | 1 | - | 1 | - | - |
| Brisk/very brisk (>3mph) | 0.69 | <0.001 | 0.69 | <0.001 | 0.99 |
| High blood pressure | 1.15 | <0.001 | 1.15 | <0.001 | 0.99 |
| Depression | 1.17 | <0.001 | 1.17 | <0.001 | 0.99 |
| Hip fracture | 1.31 | <0.001 | 1.30 | <0.001 | 0.95 |
| Parkinson’s disease | 2.35 | <0.001 | 2.37 | <0.001 | 0.94 |
| Myocardial infarction | 1.27 | <0.001 | 1.27 | <0.001 | 0.99 |
| Congestive heart failure | 1.76 | <0.001 | 1.77 | <0.001 | 0.96 |
| Stroke/Transient Ischemic Attack | 1.19 | <0.001 | 1.19 | <0.001 | 0.98 |
| Emphysema/Asthma | 1.29 | <0.001 | 1.28 | <0.001 | 0.94 |
| Diabetes | 1.36 | <0.001 | 1.37 | <0.001 | 0.97 |
| Dementia | 3.07 | <0.001 | 3.08 | <0.001 | 0.98 |
| Kidney disease | 1.28 | 0.01 | 1.31 | 0.01 | 0.87 |
| Cancer* | 1.45 | <0.001 | 1.45 | <0.001 | 0.95 |
| Age at menopause (years), <45 | 1.01 | 0.74 | 1.01 | 0.70 | 0.98 |
| 45-49 | 1 | - | 1 | - | - |
| 50-54 | 0.93 | 0.003 | 0.93 | 0.004 | 0.99 |
| 55+ | 0.87 | 0.002 | 0.87 | 0.002 | 0.97 |
| Mammogram in past 2 years. No | 1 | - | 1 | - | - |
| Yes | 0.75 | <0.001 | 0.75 | <0.001 | 0.99 |
| C-index (95% CI) | 0.795  (0.791-0.800) |  | 0.796  (0.791-0.800) |  |  |
| AIC | 188837.8 |  | 188783.9 |  |  |

1. Excludes patients with missing data on a variable.
2. The regression coefficients from the competing risk regression model and proportional hazards model were compared using the z approximation test.

eTable 6: Final competing risk regression model for predicting 10-year non-breast cancer death by age across cohorts

| 10-year non-breast cancer death | NHS Development | | | | NHS Validation | | | | BWHS | | | |
| --- | --- | --- | --- | --- | --- | --- | --- | --- | --- | --- | --- | --- |
|  | 55-74 | | 75+ | | 55-74 | | 75+ | | 55-74 | | 75+ | |
|  | HR  n=34,292 | P  value | HR  n=13,810 | P  value | HR  n=17,056 | P value | HR  n=7.032 | P value | HR  n=13,784 | P value | HR  n=1,217 | P  value |
| N for that outcome | 3,778 |  | 5,598 |  | 1,968 |  | 2,822 |  | 1,154 |  | 438 |  |
| Factors in our final model |  |  |  |  |  |  |  |  |  |  |  |  |
| Age (per age in year increase), mean (SD) | 1.11 | <0.001 | 1.10 | <0.001 | 1.11 | <0.001 | 1.12 | <0.001 | 1.09 | <0.001 | 1.12 | <0.001 |
| Highest self-reported Body Mass Index (BMI) in past 10 years: <20 kg/m^2^ | 1.54 | <0.001 | 1.66 | <0.001 | 1.72 | <0.001 | 1.50 | <0.001 | 2.75 | 0.001 | 2.59 | 0.05 |
| 20-22.4 kg/m^2^ | 1.14 | 0.05 | 1.16 | 0.003 | 1.23 | 0.02 | 1.05 | 0.46 | 0.95 | 0.79 | 2.00 | 0.01 |
| 22.5-24.9 kg/m^2^ | 1 | - | 1 | - | 1 | - | 1 | - | 1 | - | 1 | - |
| 25-29.9 kg/m^2^ | 0.91 | 0.06 | 0.92 | 0.04 | 0.89 | 0.09 | 0.87 | 0.01 | 0.74 | 0.01 | 1.08 | 0.64 |
| 30-34.9 kg/m^2^ | 0.86 | 0.01 | 0.90 | 0.02 | 0.83 | 0.02 | 0.80 | <0.001 | 0.78 | 0.04 | 1.08 | 0.67 |
| 35-39.9 kg/m^2^ | 0.88 | 0.07 | 0.94 | 0.35 | 0.91 | 0.32 | 0.93 | 0.43 | 0.93 | 0.61 | 1.04 | 0.85 |
| 40+ kg/m^2^ | 0.91 | 0.24 | 1.31 | 0.003 | 0.94 | 0.56 | 0.94 | 0.64 | 1.15 | 0.29 | 1.47 | 0.10 |
| Average alcohol use per day (highest average use in past 10 years) : None | 1 | - | 1 | - | 1 | - | 1 | - | 1 | - | 1 | - |
| 1-4.9 gram/day | 0.87 | 0.001 | 0.86 | <0.001 | 0.89 | 0.05 | 1.00 | 0.99 | 0.90 | 0.16 | 0.97 | 0.80 |
| 5-14.9 gram/day | 0.95 | 0.28 | 0.89 | 0.003 | 0.86 | 0.03 | 0.95 | 0.32 | 0.97 | 0.75 | 0.93 | 0.69 |
| 15+ gram/day | 1.03 | 0.50 | 1.05 | 0.29 | 0.97 | 0.67 | 0.95 | 0.45 | 1.23 | 0.03 | 1.44 | 0.04 |
| Cigarette use: Never | 1 | - | 1 | - | 1 | - | 1 | - | 1 | - | 1 | - |
| Current | 2.70 | <0.001 | 2.18 | <0.001 | 2.77 | <0.001 | 1.88 | <0.001 | 2.77 | <0.001 | 2.17 | <0.001 |
| Past | 1.38 | <0.001 | 1.33 | <0.001 | 1.46 | <0.001 | 1.20 | <0.001 | 1.45 | <0.001 | 1.30 | 0.01 |
| Limited from walking several blocks: Not at all | 1 | - | 1 | - | 1 | - | 1 | - | - | - | - | - |
| A little/a lot | 1.73 | <0.001 | 1.46 | <0.001 | 1.57 | <0.001 | 1.49 | <0.001 | - | - | - | - |
| Limited in bathing/dressing oneself: Not at all | 1 | - | 1 | - | 1 | - | 1 | - | - | - | - | - |
| A little or a lot | 1.65 | <0.001 | 1.41 | <0.001 | 1.75 | <0.001 | 1.40 | <0.001 | - | - | - | - |
| Walking pace: Unable to walk | 1.54 | <0.001 | 1.37 | <0.001 | 1.55 | <0.001 | 1.36 | <0.001 | - | - | - | - |
| Slow or average (<3mph) | 1 | - | 1 | - | 1 | - | 1 | - | 1 | - | 1 | - |
| Brisk/very brisk (>3mph) | 0.64 | <0.001 | 0.81 | <0.001 | 0.72 | <0.001 | 0.80 | 0.005 | 0.76 | <0.001 | 0.60 | 0.001 |
| High Blood pressure | 1.13 | 0.001 | 1.16 | <0.001 | 1.15 | 0.007 | 1.24 | <0.001 | 1.10 | 0.20 | 1.35 | 0.03 |
| Depression | 1.16 | <0.001 | 1.17 | <0.001 | 1.15 | 0.009 | 1.18 | <0.001 | 1.09 | 0.24 | 1.12 | 0.40 |
| Hip Fracture | 1.26 | 0.03 | 1.35 | <0.001 | 1.37 | 0.03 | 1.34 | <0.001 | 1.49 | 0.15 | 0.85 | 0.63 |
| Parkinson’s Disease | 2.78 | <0.001 | 2.25 | <0.001 | 2.53 | <0.001 | 2.23 | <0.001 | 6.38 | <0.001 | 3.14 | <0.001 |
| Myocardial Infarction | 1.22 | 0.002 | 1.28 | <0.001 | 1.35 | <0.001 | 1.06 | 0.39 | 1.15 | 0.25 | 1.04 | 0.83 |
| Congestive heart failure | 2.12 | <0.001 | 1.66 | <0.001 | 2.30 | <0.001 | 1.89 | <0.001 | 2.25 | <0.001 | 1.89 | <0.001 |
| Stroke/Transient Ischemic Attack | 1.18 | 0.005 | 1.21 | <0.001 | 1.31 | <0.001 | 1.21 | <0.001 | 1.65 | <0.001 | 1.35 | 0.07 |
| Emphysema/Asthma | 1.24 | <0.001 | 1.32 | <0.001 | 1.22 | <0.001 | 1.31 | <0.001 | 1.07 | 0.34 | 0.92 | 0.52 |
| Diabetes | 1.45 | <0.001 | 1.29 | <0.001 | 1.32 | <0.001 | 1.28 | <0.001 | 1.48 | <0.001 | 1.41 | 0.001 |
| Dementia | 3.96 | <0.001 | 2.92 | <0.001 | 3.74 | <0.001 | 2.72 | <0.001 | 1.87 | 0.15 | 1.61 | 0.27 |
| Kidney Disease | 1.46 | 0.04 | 1.20 | 0.30 | 1.03 | 0.89 | 1.36 | 0.25 | 2.53 | <0.001 | 1.80 | 0.01 |
| Cancer* | 1.77 | <0.001 | 1.29 | <0.001 | 1.74 | <0.001 | 1.28 | <0.001 | 2.65 | <0.001 | 2.44 | <0.001 |
| Age at menopause (years), <45 | 1.02 | 0.72 | 1.01 | 0.86 | 0.99 | 0.92 | 0.92 | 0.30 | 0.97 | 0.74 | 1.11 | 0.48 |
| 45-49 | 1 | - | 1 | - | 1 | - | 1 | - | 1 | - | 1 | - |
| 50-54, | 0.91 | 0.02 | 0.95 | 0.11 | 0.89 | 0.03 | 0.93 | 0.10 | 0.85 | 0.04 | 1.17 | 0.26 |
| 55+ | 0.81 | 0.003 | 0.92 | 0.17 | 0.83 | 0.05 | 0.90 | 0.20 | 0.96 | 0.75 | 1.07 | 0.72 |
| Mammogram in past 2 years: No | 1 | - | 1 | - | 1 | - | 1 | - | 1 | - | 1 | - |
| Yes | 0.69 | <0.001 | 0.78 | <0.001 | 0.63 | <0.001 | 0.80 | <0.001 | 0.61 | <0.001 | 0.63 | <0.001 |
| C-index (95% CI) | 0.765 (0.758-0.773) | | 0.699 (0.692-0.705) | | 0.762 (0.751-0.773) | | 0.698 (0.689-0.708) | | 0.752 (0.737-0.766) | | 0.687 (0.661-0.712) | |
| C-index (95% CI) when using risk factor regression coefficients from the NHS cohort in the BWHS cohort | - | | - | | 0.760 (0.749-0.770) | | 0.696 (0.686-0.706) | | 0.735 (0.721-0.750) | | 0.671 (0.645-0.696) | |

eTable 7 Calibration table for predicting 10-year non-breast cancer death and 5-year breast cancer risk by age.^a,b,c^

| Predicting 10-year non-breast cancer death | | | | | | | | | | | | |
| --- | --- | --- | --- | --- | --- | --- | --- | --- | --- | --- | --- | --- |
| 55-74 | | | | | | | | | | | | |
|  |  | NHS Development Cohort n=34,292^c^ | | | NHS Validation Cohort  n=17,056^c^ | | | | Black Women’s Health Study  n=13,784^c^ | | | |
| Risk Group^d^ | Time (years) | Deaths (%) | Expected Survival  (using CRR) | Observed Survival | Deaths (%) | Expected Survival  (using CRR) | Observed Survival | Expected/Observed (E/O) | Deaths (%) | Expected Survival  (using CRR) | Observed Survival | Expected/  Observed (E/O) |
| 1 | 5 |  | 0.99 | 0.99 |  | 0.99 | 0.99 | 1.00 |  | 0.99 | 0.99 | 1.00 |
|  | 10 | 251 (2.6) | 0.96 | 0.98 | 126 (2.7) | 0.96 | 0.98 | 0.98 | 74 (2.2) | 0.96 | 0.98 | 0.98 |
| 2 | 5 |  | 0.98 | 0.99 |  | 0.98 | 0.98 | 1.00 |  | 0.98 | 0.98 | 1.00 |
|  | 10 | 550 (5.7) | 0.92 | 0.95 | 303 (6.3) | 0.93 | 0.94 | 0.99 | 220 (5.2) | 0.92 | 0.95 | 0.97 |
| 3 | 5 |  | 0.96 | 0.97 |  | 0.96 | 0.96 | 1.00 |  | 0.96 | 0.97 | 0.99 |
|  | 10 | 968 (11.5) | 0.87 | 0.89 | 506 (12.0) | 0.86 | 0.89 | 0.97 | 283 (8.1) | 0.87 | 0.92 | 0.95 |
| 4 | 5 |  | 0.92 | 0.93 |  | 0.92 | 0.93 | 0.99 |  | 0.92 | 0.94 | 0.98 |
|  | 10 | 1140 (23.4) | 0.76 | 0.78 | 559 (23.3) | 0.76 | 0.78 | 0.97 | 363 (17.8) | 0.76 | 0.83 | 0.92 |
| 5 | 5 |  | 0.78 | 0.80 |  | 0.81 | 0.78 | 1.04 |  | 0.79 | 0.84 | 0.94 |
|  | 10 | 869 (48.2) | 0.53 | 0.54 | 474 (52.4) | 0.49 | 0.49 | 1.00 | 214 (37.4) | 0.50 | 0.64 | 0.78 |
| 75+ | | | | | | | | | | | | |
|  |  | NHS Development Cohort n=13,810^c^ | | | NHS Validation Cohort  n=7,032^c^ | | | | Black Women’s Health Study  n=1,217^c^ | | | |
| Risk Group^d^ | Time (years) | Deaths (%) | Expected Survival  (using CRR) | Observed Survival | Deaths (%) | Expected Survival  (using CRR) | Observed Survival | Expected/Observed (E/O) | Deaths (%) | Expected Survival  (using CRR) | Observed Survival | Expected/  Observed (E/O) |
| 1 | 5 |  | - | - |  | - | - | - |  | - | - | - |
|  | 10 | 0 (0.0) | - | - | 0 (0.0) | - | - | - | 0 (0.0) | - | - | - |
| 2 | 5 |  | 0.97 | 0.93 |  | - | - | - |  | - | - | - |
|  | 10 | 3 (7.3) | - | - | 2 (10.0) | - | - | - | 0 (0.0) | - | - | - |
| 3 | 5 |  | 0.95 | 0.96 |  | 0.95 | 0.96 | 0.99 |  | - | - | - |
|  | 10 | 165 (13.8) | 0.85 | 0.87 | 88 (13.5) | 0.85 | 0.87 | 0.98 | 1 (8.3) | - | - | - |
| 4 | 5 |  | 0.91 | 0.94 |  | 0.92 | 0.95 | 0.97 |  | 0.91 | 0.93 | 0.98 |
|  | 10 | 1180 (24.8) | 0.74 | 0.78 | 613 (25.7) | 0.74 | 0.76 | 0.97 | 60 (19.7) | 0.70 | 0.82 | 0.85 |
| 5 | 5 |  | 0.72 | 0.80 |  | 0.72 | 0.80 | 0.90 |  | 0.74 | 0.84 | 0.88 |
|  | 10 | 4250 (54.4) | 0.46 | 0.48 | 2119 (53.3) | 0.46 | 0.49 | 0.94 | 377 (41.8) | 0.42 | 0.60 | 0.70 |

| Predicting 5-year invasive breast cancer risk | | | | | | | | | | | | |
| --- | --- | --- | --- | --- | --- | --- | --- | --- | --- | --- | --- | --- |
| 55-74 | | | | | | | | | | | | |
|  |  | NHS Development Cohort n=28,488^c^ | | | NHS Validation Cohort n=14,264^c^ | | | | Black Women’s Health Study n=12,216 | | | |
| Risk Group | Time (years) | Invasive breast cancers (%) | Expected Survival  (using CRR) | Observed Survival | Invasive breast cancers (%) | Expected Survival  (using CRR) | Observed Survival | Expected/Observed (E/O) | Invasive breast cancers (%) | Expected Survival  (using CRR) | Observed Survival | Expected/  Observed (E/O) |
| 1 | 5 | 63 (1.1) | 0.99 | 0.99 | 29 (1.0) | 0.99 | 0.99 | 1.00 | 38 (1.2) | 0.99 | 0.99 | 1.00 |
| 2 | 5 | 90 (1.6) | 0.98 | 0.99 | 40 (1.3) | 0.99 | 0.99 | 1.00 | 36 (1.5) | 0.98 | 0.99 | 0.99 |
| 3 | 5 | 90 (1.6) | 0.98 | 0.99 | 60 (2.0) | 0.98 | 0.98 | 1.00 | 35 (1.5) | 0.98 | 0.99 | 0.99 |
| 4 | 5 | 113 (2.0) | 0.98 | 0.98 | 54 (2.0) | 0.98 | 0.98 | 1.00 | 42 (1.9) | 0.98 | 0.98 | 1.00 |
| 5 | 5 | 183 (3.3) | 0.97 | 0.97 | 92 (3.3) | 0.97 | 0.97 | 1.00 | 46 (2.2) | 0.97 | 0.98 | 0.99 |
| 75+ | | | | | | | | | | | | |
|  |  | NHS Development Cohort n=9,140^c^ | | | NHS Validation Cohort n=4,716^c^ | | | | Black Women’s Health Study n=1,031 | | | |
| Risk Group | Time (years) | Invasive breast cancers (%) | Expected Survival  (using CRR) | Observed Survival | Invasive breast cancers (%) | Expected Survival  (using CRR) | Observed Survival | Expected/Observed (E/O) | Invasive breast cancers (%) | Expected Survival  (using CRR) | Observed Survival | Expected/  Observed (E/O) |
| 1 | 5 | 17 (1.1) | 0.99 | 0.99 | 11 (1.4) | 0.99 | 0.99 | 1.00 | 2 (1.2) | - | - | - |
| 2 | 5 | 32 (1.8) | 0.99 | 0.98 | 12 (1.3) | 0.98 | 0.99 | 0.99 | 4 (2.0) | - | - | - |
| 3 | 5 | 22 (1.2) | 0.98 | 0.99 | 14 (1.4) | 0.98 | 0.99 | 0.99 | 4 (2.0) | 0.98 | 0.98 | 1.00 |
| 4 | 5 | 37 (1.9) | 0.98 | 0.98 | 22 (2.2) | 0.98 | 0.98 | 1.00 | 8 (3.2) | 0.98 | 0.97 | 1.01 |
| 5 | 5 | 65 (3.3) | 0.96 | 0.97 | 31 (3.1) | 0.97 | 0.97 | 1.00 | 8 (3.7) | 0.97 | 0.96 | 1.01 |

1. Abbreviations: CRR=competing risk regression, CIF=cumulative incidence function, NHS=Nurses’ Health Study, BWHS=Black Women’s Health Study.
2. Calibration of the model in predicting non-BC death was assessed by estimating the ratio of the expected survival (1-CIF for non-BC death from our CRR model) to the observed survival (1-the observed CIF computed using the nonparametric estimation of CIF) at 5 and 10 years within risk quintiles. Similar methods were used to test calibration of the model in predicting BC.
3. These analyses include women with complete data. When predicting breast cancer, women with a history of cancer were further excluded.
4. Risk groups were defined by quintiles of the NHS prognostic index for each outcome which was calculated using NHS regression coefficients.

eTable 8: Cause-Specific Proportional Hazards Regression Model in predicting 5-year breast cancer risk in NHS development cohort

| Predicting 5-year risk of breast cancer | Competing Risk Regression (CRR) | | Cause-specific Proportional Hazards regression | |  |
| --- | --- | --- | --- | --- | --- |
| Outcome being predicted | HR  n=37,628 | P value | HR  n=37,628 | P value | P value for difference in risk factor regression coefficients from CRR model |
| N for that outcome | 712 |  | 712 |  |  |
| Factors in our final model |  |  |  |  |  |
| Age (per year increase) | 1.01 | 0.10 | 1.01 | 0.03 | 0.81 |
| Highest self-reported Body Mass Index (BMI) in past 10 years: (per kg/m^2^ increase) | 1.03 | <0.001 | 1.03 | <0.001 | 0.94 |
| Average alcohol use per day (highest average use in past 10 years) : None | 1 | - | 1 | - | - |
| 1-4.9 gram/day | 1.12 | 0.27 | 1.11 | 0.30 | 0.95 |
| 5-14.9 gram/day | 1.20 | 0.09 | 1.19 | 0.10 | 0.97 |
| 15+ gram/day | 1.29 | 0.02 | 1.29 | 0.02 | 0.99 |
| Age at menopause (years), <45 | 0.91 | 0.57 | 0.91 | 0.57 | 0.98 |
| 45-49 | 1 | - | 1 | - | - |
| 50-54, | 1.21 | 0.04 | 1.21 | 0.05 | 0.98 |
| 55+ | 1.32 | 0.05 | 1.32 | 0.05 | 0.98 |
| Mammogram in past 2 years\|\| No | 1 | - | 1 | - | - |
| Yes | 0.91 | 0.46 | 0.89 | 0.34 | 0.88 |
| Number of breast biopsies, None | 1 | - | 1 | - | - |
| 1 | 1.38 | <0.001 | 1.38 | <0.001 | 0.99 |
| 2+ | 1.31 | 0.16 | 1.31 | 0.16 | 0.99 |
| Age at first birth (years) and parity, Nulliparous | 1.25 | 0.23 | 1.25 | 0.23 | 0.99 |
| <25, 1-2 children | 1 | - | 1 | - | - |
| <25, 3+ children | 1.06 | 0.62 | 1.05 | 0.66 | 0.97 |
| 25-29, 1-2 children | 1.07 | 0.63 | 1.07 | 0.64 | 0.99 |
| 25-29, 3+ children | 1.04 | 0.76 | 1.03 | 0.80 | 0.98 |
| 30+, 1-2 children | 1.37 | 0.07 | 1.36 | 0.08 | 0.98 |
| 30+, 3+ children | 1.06 | 0.82 | 1.05 | 0.85 | 0.98 |
| First-degree relatives with history of breast cancer and age at diagnosis, None | 1 | - | 1 | - | - |
| 1 and age <50 | 1.43 | 0.03 | 1.42 | 0.03 | 0.99 |
| 1 and age 50+ | 1.30 | 0.02 | 1.29 | 0.02 | 0.99 |
| 2+ and at least one age <50 | 2.19 | 0.003 | 2.16 | 0.003 | 0.97 |
| 2+ and age 50+ | 2.43 | <0.001 | 2.45 | <0.001 | 0.98 |
| Postmenopausal hormone use, Never | 1 | - | 1 | - | - |
| Current estrogen + progestin user <5 years | 2.28 | 0.07 | 2.29 | 0.07 | 0.99 |
| Current estrogen + progestin user 5+ years | 2.60 | <0.001 | 2.59 | <0.001 | 0.99 |
| Current estrogen-alone user <5 years | 1.60 | 0.15 | 1.60 | 0.15 | 0.99 |
| Current estrogen-alone user 5+ years | 1.36 | 0.02 | 1.36 | 0.02 | 0.99 |
| Past estrogen +progestin user <5 years | 0.91 | 0.47 | 0.91 | 0.46 | 0.99 |
| Past estrogen +progestin user 5+ years | 1.17 | 0.17 | 1.17 | 0.18 | 0.99 |
| Past estrogen-alone user <5 years | 1.01 | 0.93 | 1.02 | 0.92 | 0.99 |
| Past estrogen-alone user 5+ years | 0.77 | 0.06 | 0.77 | 0.06 | 0.99 |
| C-index (95% CI) | 0.610  (0.589-0.631) | | 0.610  (0.589-0.631) | |  |
| AIC | 14914.7 | | 14892.0 | |  |

eTable 9: Final competing risk regression model for predicting 5-year breast cancer risk by age across cohorts.

| Predicting 5-year risk of breast cancer | NHS Development | | | | NHS Validation | | | | BWHS | | | |
| --- | --- | --- | --- | --- | --- | --- | --- | --- | --- | --- | --- | --- |
|  | 55-74 | | 75+ | | 55-74 | | 75+ | | 55-74 | | 75+ | |
|  | HR  n=28,488 | P  value | HR  n=9,140 | P value | HR  n=14,264 | P value | HR  n=4,716 | P value | HR  n=12,216 | P value | HR  n=1,031 | P value |
| N for that outcome | 539 |  | 173 |  | 275 |  | 90 |  | 197 |  | 26 |  |
| Factors in our final model |  |  |  |  |  |  |  |  |  |  |  |  |
| Age (per year increase) | 1.02 | 0.06 | 0.97 | 0.29 | 1.02 | 0.25 | 0.95 | 0.29 | 1.00 | 0.77 | 1.09 | 0.24 |
| Highest self-reported Body Mass Index (BMI) in past 10 years: (per kg/m^2^ increase) | 1.02 | 0.001 | 1.04 | 0.01 | 1.02 | 0.02 | 1.02 | 0.28 | 1.02 | 0.02 | 1.03 | 0.46 |
| Average alcohol use per day (highest average use in past 10 years) : None | 1 | - | 1 | - | 1 | - | 1 | - | 1 | - | 1 | - |
| 1-4.9 gram/day | 1.07 | 0.57 | 1.33 | 0.14 | 0.98 | 0.88 | 1.02 | 0.95 | 0.72 | 0.08 | 1.56 | 0.42 |
| 5-14.9 gram/day | 1.27 | 0.05 | 0.96 | 0.84 | 0.99 | 0.96 | 0.97 | 0.92 | 0.83 | 0.41 | 3.17 | 0.04 |
| 15+ gram/day | 1.31 | 0.04 | 1.20 | 0.43 | 0.98 | 0.91 | 1.56 | 0.14 | 1.18 | 0.47 | 2.34 | 0.14 |
| Age at menopause (years), <45 | 0.92 | 0.64 | 0.93 | 0.83 | 0.71 | 0.19 | 0.37 | 0.19 | 0.98 | 0.94 | 1.63 | 0.40 |
| 45-49 | 1 | - | 1 | - | 1 | - | 1 | - | 1 | - | 1 | - |
| 50-54, | 1.18 | 0.12 | 1.34 | 0.15 | 1.06 | 0.70 | 1.84 | 0.04 | 1.25 | 0.23 | 0.62 | 0.39 |
| 55+ | 1.17 | 0.33 | 1.88 | 0.03 | 0.98 | 0.93 | 1.98 | 0.11 | 1.07 | 0.83 | 1.34 | 0.64 |
| Mammogram in past 2 years,\| No | 1 | - | 1 | - | 1 | - | 1 | - | 1 | - | 1 | - |
| Yes | 0.84 | 0.21 | 1.08 | 0.74 | 0.87 | 0.48 | 0.86 | 0.61 | 0.67 | 0.03 | 0.71 | 0.49 |
| Number of breast biopsies, None | 1 | - | 1 | - | 1 | - | 1 | - | 1 | - | 1 | - |
| 1 | 1.50 | <0.001 | 1.06 | 0.75 | 1.54 | 0.001 | 1.39 | 0.17 | 1.29 | 0.12 | 1.57 | 0.45 |
| 2+ | 1.26 | 0.28 | 1.51 | 0.30 | 1.63 | 0.07 | 2.05 | 0.17 | 1.00 | 0.99 | 2.75 | 0.04 |
| Age at first birth (years) and parity, Nulliparous | 1.31 | 0.18 | 1.27 | 0.62 | 1.29 | 0.40 | 0.18 | 0.10 | 1.04 | 0.87 | 1.69 | 0.49 |
| <25, 1-2 children | 1 | - | 1 | - | 1 | - | 1 | - | 1 | - | 1 | - |
| <25, 3+ children | 0.94 | 0.62 | 1.73 | 0.11 | 1.24 | 0.27 | 0.70 | 0.35 | 1.07 | 0.72 | 0.95 | 0.93 |
| 25-29, 1-2 children | 0.99 | 0.97 | 1.64 | 0.19 | 1.18 | 0.45 | 0.82 | 0.64 | 1.30 | 0.24 | 1.96 | 0.38 |
| 25-29, 3+ children | 1.00 | 0.99 | 1.37 | 0.37 | 1.06 | 0.82 | 0.84 | 0.64 | 1.46 | 0.34 | 2.36 | 0.32 |
| 30+, 1-2 children | 1.43 | 0.07 | 1.53 | 0.31 | 2.07 | 0.007 | 1.41 | 0.44 | 1.24 | 0.44 | 3.14 | 0.15 |
| 30+, 3+ children | 1.09 | 0.79 | 1.33 | 0.54 | 0.99 | 0.99 | 0.18 | 0.10 | 2.40 | 0.22 | 7.49 | 0.11 |
| First-degree relatives with history of breast cancer and age at diagnosis*, None | 1 | - | 1 | - | 1 | - | 1 | - | 1 | - | 1 | - |
| 1 and age <50 | 1.46 | 0.04 | 1.32 | 0.40 | 1.18 | 0.57 | 1.60 | 0.28 | 1.16 | 0.69 | 1.84 | 0.46 |
| 1 and age 50+ | 1.32 | 0.03 | 1.25 | 0.31 | 1.23 | 0.24 | 1.53 | 0.14 | 1.21 | 0.31 | 2.78 | 0.05 |
| 2+ and at least one age <50 | 1.71 | 0.14 | 3.05 | 0.005 | 3.23 | <0.001 | 3.28 | 0.02 | 1.81 | 0.41 | - | - |
| 2+ and age 50+ | 2.50 | 0.005 | 2.26 | 0.03 | 1.28 | 0.68 | 1.91 | 0.29 | 0.44 | 0.41 | - | - |
| Postmenopausal hormone use*, Never | 1 | - | 1 | - | 1 | - | 1 | - | 1 | - | 1 | - |
| Current estrogen + progestin user <5 years | 2.61 | 0.04 | - | - | 1.93 | 0.35 | - | - | 2.47 | 0.13 | - | - |
| Current estrogen + progestin user 5+ years | 2.32 | <0.001 | 4.83 | <0.001 | 3.12 | <0.001 | 0.94 | 0.95 | 2.09 | 0.15 | 14.87 | 0.01 |
| Current estrogen-alone user <5 years | 1.76 | 0.09 | - | - | 1.21 | 0.75 | 3.17 | 0.25 | 1.93 | 0.12 | - | - |
| Current estrogen-alone user 5+ years | 1.20 | 0.24 | 1.96 | 0.006 | 1.34 | 0.16 | 1.17 | 0.70 | 1.10 | 0.75 | 0.42 | 0.43 |
| Past estrogen +progestin user <5 years | 0.88 | 0.37 | 1.05 | 0.85 | 0.87 | 0.49 | 0.63 | 0.28 | 1.14 | 0.55 | 0.25 | 0.24 |
| Past estrogen +progestin user 5+ years | 1.14 | 0.33 | 1.19 | 0.51 | 1.15 | 0.45 | 1.22 | 0.58 | 1.66 | 0.09 | 0.79 | 0.77 |
| Past estrogen-alone user <5 years | 1.09 | 0.67 | 0.96 | 0.88 | 0.61 | 0.17 | 1.05 | 0.89 | 1.08 | 0.73 | 0.99 | 0.99 |
| Past estrogen-alone user 5+ years | 0.73 | 0.07 | 0.87 | 0.56 | 0.94 | 0.76 | 1.45 | 0.20 | 0.87 | 0.58 | 0.26 | 0.08 |
| C-index (95% CI) | 0.615  (0.591-0.639) | | 0.650  (0.608-0.692) | | 0.644  (0.612-0.677) | | 0.683  (0.631-0.735) | | 0.619  (0.583-0.656) | | 0.753  (0.660-0.846) | |
| C-index (95% CI) when using risk factor regression coefficients from the NHS cohort in the BWHS cohort | - | | - | | 0.618  (0.585-0.650) | | 0.596  (0.534-0.657) | | 0.566  (0.526-0.606) | | 0.614  (0.506-0.722) | |

* In the 75+ age group in BWHS cohort there was complete separation between breast cancer cases and having 2+ first-degree relatives with history of breast cancer. In the 75+ age group in both the NHS development cohort and BWHS cohort there was complete separation between breast cancer cases and current estrogen+progestin and estrogen-alone users with <5 years of duration. In the 75+ age group in the NHS validation cohort there was complete separation between breast cancer cases and current estrogen+progestin users with <5 years of duration.

**eFigure 1:** Sample Population

Women aged 55+ in the Nurses’ Health Study (NHS) and Black Women’s Health Study (BWHS)

Died before start of follow-up^a^

Did not return questionnaire or chose not to participate^b^

History of breast cancer

Excluded

NHS

n=121,529

18,275

103,254

12,474

83,330

0

Age 55-74

n= 59,684

Age 75+

n= 23,646

BWHS

n=59,001

4,407

10,063

54,594

10,993

43,601

17,380

25,372

Age 55-74

n=15,946

Age 75+

n= 1,434

<55 years or premenopausal^c^

90,780

90,780

7,450

849

18,229

1. NHS: died before 2004; BWHS: died before 2009
2. NHS: Did not return 2004 questionnaire; BWHS: Did not return 2009 questionnaire;
3. We excluded those who were less than 55 years of age or premenopausal at start of follow-up.

**Appendix A: Description of the Nurses’ Health Study (NHS) and Black Women’s Health Study (BWHS)**

Nurses’ Health Study:

The NHS is a longitudinal study of 121,700 US female nurses (97% non-Hispanic white), ages 30-55 in 1976, living in 11 of the most populous US states (California, Connecticut, Florida, Maryland, Massachusetts, Michigan, New Jersey, New York, Ohio, Pennsylvania, or Texas) at baseline. Nurses were chosen to participate because of the higher accuracy of health information that they could provide and potentially greater willingness to participate in a longitudinal health study than a general cohort (71.5% of the 170,000 female nurses invited to participate returned baseline questionnaires). At baseline and in biennial follow-ups, participants provide detailed lifestyle, demographic, and medical history information through mailed questionnaires. Follow-up questionnaires are mailed in June of even-numbered years and non-responders are sent a second mailing in September. A third and fourth questionnaire are sent to those who still have not responded. A fifth mailing of a shorter questionnaire with key exposures and the list of major illnesses is finally sent. Since 1982, women who have not responded to any of the five mailings are telephoned for follow-up. Using these procedures, ~90% of those living complete a questionnaire every 2 years.^1^ All dates in NHS are recorded as months from the year 1900.

Black Women’s Health Study:

The BWHS is an ongoing prospective follow-up study of self-identified Black women in the U.S. It was designed to gather information on many conditions that affect black women including breast cancer, hypertension, colon cancer, diabetes, lupus, etc. BWHS began in 1995 when women 21-69 years of age were enrolled through postal questionnaires mailed to subscribers of Essence magazine, to members of several professional organizations (e.g., the National Education Association, federal government employees) and to friends and relatives of early responders. The women were from across the U.S. with over 80% from California, Georgia, Illinois, Indiana, Louisiana, Maryland, Massachusetts, Michigan, New Jersey, New York, South Carolina, Virginia, and the District of Columbia. The baseline questionnaire collected information on demographics, medical and reproductive history, height, weight, smoking, and physical activity, use of postmenopausal hormone therapy (duration, type or preparation, last use), menopausal status, and medical history. Overall, 59,064 women made up the cohort; 27% lived in the Northeast, 29% in the south, 23% in the Midwest, and 21% in the west. The population is 100% non-Hispanic black. Participants are followed through biennial postal questionnaires. Follow-up questionnaires collect updated information on weight, smoking, physical activity, incident disease, reproductive factors, and health conditions and as well as new information on other health factors. The institutional review boards of Boston University Medical Center approved the BWHS. BWHS study questionnaires (which have been conducted biennially since 1995) are available on BWHS’s website (<https://www.bu.edu/bwhs/>).

**Appendix B: Data Dictionary: Nurses’ Health Study (NHS)**

***Breast cancer***

We included confirmed cases of invasive breast cancer diagnosed during our study period. Women with in situ diagnoses during follow-up were censored. NHS women entered our study at the time that they returned their 2004 questionnaire which could be returned anytime between June 2004 and May 2006. We followed women up to November 2018, the month of most recent confirmed deaths.

***Death***

Most deaths are reported by participants’ next of kin or by postal authorities. These reports are supplemented by searches of the National Death Index (NDI); Using these methods >98% of deaths have been identified. Deaths were counted if they occurred between the 2004 questionnaire return date, and November 2018 (most recent deaths confirmed by NHS).

***Breast cancer death/ Non-breast cancer death***

A death was considered due to breast cancer if it had an NHS confirmed ICD-8 cause of death code of 174. More recent ICD-9 and 10 codes for death are converted to ICD-8 codes. Otherwise, a death was considered a non-breast cancer death.

***Age***

We measured age at 2004 questionnaire return date by calculating the number of months between a participant’s birth month and year and month and year of return of the 2004 questionnaire. The number of months was divided by 12 to determine age in years.

***Factors considered in the model:***

***Age of death of biological mother/father:***

In the 1988 questionnaire participants were asked whether their biological mother and father were still alive, and if not, at what age they died, respectively. We combined these questions to create a variable for age of death of biological mother or father (<50, 50-59, 60-69, 70-79, 80+, alive). The younger age of death between the mother and father was used for each participant.

**Health Behaviors:**

***Body Mass Index (BMI) (kg/m^2^)***

To calculate BMI, we used the highest self-reported weight within the 10 years before start of follow up. Height was self-reported in 1976. Weight has been assessed on every questionnaire since 1976. We chose to use the highest weight reported in the past 10 years since older women tend to lose weight as they approach death as a result of illness and frailty. In fact, women >75 in our cohort had their peak BMI 10 years before the 2004 questionnaire whereas women 55-74 had their peak BMI in 2000 (4 years before the 2004 questionnaire).

***Alcohol consumption, maximum gram/day in the past 10 years (none, 1-<5 gm/day, 5-<15 gm/day, 15+ gm/day)***

Alcohol consumption was collected in years 80, 84, 86, 90, 94, 98, and 02. We used the derived alcohol variable of average grams/day over the past year. Since alcohol use may decline as women become sick or frail, we used the maximum average grams/day reported in the past 10 years. These data were obtained from the 1998 and 02 questionnaires. We considered participants who did not respond to questions on alcohol consumption in 98 and 02 to have missing values. In general, 15+ grams/day suggest a drink per day while 5-<15 grams per day suggest 2-5 drinks per week and 1-<5 grams/day suggest <2 drinks per week but more than none.

***Cigarette use (never, past, current)***

Cigarette use was collected on every survey. We used the derived variable that derived months since the participants quit smoking (if they quit smoking), and whether a participant currently smoked or never smoked. We categorized women who quit smoking as former users. When data were missing from the 2004 questionnaire on cigarette use we used data from the 2002 questionnaire.

***Use of sugary drinks***

Participants were given a food frequency questionnaire every 4 years which included 3 questions about how many servings of sugary drinks they consumed. These included carbonated beverages with sugar and caffeine, carbonated beverages with sugar (caffeine-free), and non-carbonated beverages, punch, lemonade, sugared iced tea/other. If participants consumed at least one of these beverages per week, we considered them sugary drink users. We used participant response to the 2002 questionnaire or in 1998 or 1994 when data from 2002 were missing.

**Function:**

***Amount of bodily pain – None/mild vs. moderate/severe/very severe***

Participants were asked on the 1996 and 2000 questionnaires how much bodily pain they had during the past 4 weeks (none, very mild, mild, moderate, severe, very severe). We combined none to mild into one category, and moderate to very severe into another category, resulting in 2 total categories. We used responses from 2000, and if missing, from 1996.

***Difficulty with balance***

Participants were asked in the 90, 96 and 04 questionnaires whether they had difficulty with their balance (yes vs. no). We used participant response to the 1996 questionnaire or in prior years when data from 2004 were missing.

***Fell at least once in past year***

Participants were asked in the 98, 00 and 04 questionnaires the number of times they had fallen to the ground in the past year. This variable indicates whether a participant fell at least once in that year (vs. no falls). We used participant response to the 2000 questionnaire or in prior years when data from 2004 were missing.

***Flights of stairs climbed daily (2 flights or less, 3-4, 5-9, 10-14, 15+)***

Participants were asked in the 96, 98, 00 and 04 questionnaires how many flights of stairs they climb daily (2 flights or less, 3-4, 5-9, 10-14, 15+). We used participant response to the 2000 questionnaire or in prior years when data from 2004 were missing.

***Limited bathing/dressing***

Participants who completed the long version of the NHS questionnaire in 92, 96, 00, and 04 were asked if their health now limits them in bathing or dressing themselves. Response categories included not at all limited, a little limited or a lot limited. We grouped a little limited with a lot limited since the hazard ratios for non-breast cancer death associated with these levels were similar. We used participant response to the 2000 questionnaire or in prior years when data from 2004 were missing.

***Limited bending/kneeling/stooping***

Participants who completed the long version of the NHS questionnaire in 92, 96, 00, and 04 were asked if their health now limits them from bending, kneeling or stooping. Response categories included not at all limited, a little limited or a lot limited. We grouped a little limited with a lot limited since the hazard ratios for death associated with these levels were similar. We used participant response to the 2000 questionnaire or in prior years when data from 2004 were missing.

***Limited climbing several flights of stairs***

Participants who completed the long version of the NHS questionnaire in 92, 96, 00, and 04 were asked if their health now limits them from climbing several flights of stairs. Response categories included not at all limited, a little limited or a lot limited. We grouped a little limited with a lot limited since the hazard ratios for death associated with these levels were similar. We used participant response to the 2000 questionnaire or in prior years when data from 2004 were missing.

***Limited lifting/carrying groceries***

Participants who completed the long version of the NHS questionnaire in 92, 96, 00, and 04 were asked if their health now limits them from lifting or carrying groceries. Response categories included not at all limited, a little limited or a lot limited. We grouped a little limited with a lot limited since the hazard ratios for death associated with these levels were similar. We used participant response to the 2000 questionnaire or in prior years when data from 2004 were missing.

***Limited walking several blocks***

Participants who completed the long version of the NHS questionnaire in 92, 96, 00, and 04 were asked if their health now limits them from walking several blocks. Response categories included not at all limited, a little limited or a lot limited. We grouped a little limited with a lot limited since the hazard ratios for death associated with these levels were similar. We used participant response to the 2000 questionnaire or in prior years when data from 2004 were missing.

***Limited walking several blocks***

Participants who completed the long version of the NHS questionnaire in 92, 96, 00, and 04 were asked if their health now limits them from walking several blocks. Response categories included not at all limited, a little limited or a lot limited. We grouped a little limited with a lot limited since the hazard ratios for death associated with these levels were similar. We used participant response to the 2000 questionnaire or in prior years when data from 2004 were missing.

***Limited in vigorous activity***

Participants who completed the long version of the NHS questionnaire in 92, 96, 00, and 04 were asked if their health now limits them from vigorous activities such as running, lifting heavy objects, or participating in strenuous sports. Response categories included not at all limited, a little limited or a lot limited. We grouped a little limited with a lot limited since the hazard ratios for death associated with these levels were similar. We used participant response to the 2000 questionnaire or in prior years when data from 2004 were missing.

***Limited in moderate activity***

Participants who completed the long version of the NHS questionnaire in 92, 96, 00, and 04 were asked if their health now limits them from moderate activities such as moving a table, pushing a vacuum cleaner, bowling, or playing golf. Response categories included not at all limited, a little limited or a lot limited. We grouped a little limited with a lot limited since the hazard ratios for death associated with these levels were similar. We used participant response to the 2000 questionnaire or in prior years when data from 2004 were missing.

***Physical activity, maximum average METs/week in the past 10 years (0-2.9, 3-8.9, 9-26.9, 27+)***

We used the derived variable of average METs/week during the past year from surveys 96, 98, 00, and 04. We used the maximum level reported from these questionnaires since as women age and become more frail they tend to be less active. Based on previous studies using these data, we set implausible values (125+ METs/week) to missing. This variable was also considered for prediction of breast cancer and breast cancer death.

***Physical health limiting work***

Participants were asked in separate questions on the 1996 and 2000 questionnaires whether they cut down the amount of time they spent on work, or if they had difficulty performing work due to physical health. We combined these questions – if a participant answered yes to either of them we counted them as experiencing difficulty working due to physical health. We used responses from 2000, and if missing, from 1996.

***Use of cane/walker***

Participants were asked whether they usually use a cane or walker in the 2004 questionnaire only.

***Walking pace***

Participants were asked in the 96, 98, 00, and 04 questionnaires about their walking pace outdoors. Response categories included: easy/casual (less than 2 mph), normal/average (2-2.9 mph), brisk (3-3.9) mph, very brisk/striding (4+ mph), unable to walk. We combined easy/casual and normal/average into one level, and brisk and very brisk into one level resulting in three levels: unable to walk, easy/average, and brisk/very brisk. We used participant response to the 2000 questionnaire or in prior years when data from 2004 were missing.

***Comorbidities***

All comorbidities were self-reported as physician-diagnosed. We chose not to use only cases confirmed through medical record review since the rate of disease was very comparable across both methods. Also, in disseminating the model, women will often likely answer the questions on risk factors without help from a health care professional.

All comorbidities are defined in detail below:

***Amyotrophic Lateral Sclerosis (ALS)***

Physician-diagnosed ALS was assessed starting with the 1976 questionnaire.

***AIDS***

AIDS, as indicated by ICD-8 code 079, was assessed starting with the 1978 questionnaire.

***Angina***

Physician-diagnosed angina was assessed starting with the 1976 questionnaire.

***Cancer***

Cancer was assessed starting with the 1976 questionnaire. All cancers except non-melanoma skin cancer and breast cancer were included.

***Chronic Kidney Disease***

We defined chronic kidney disease as any one of ICD-8 codes 581 (nephrotic syndrome), 582 (chronic glomerulonephritis), 583 (nephritis and nephropathy not specified as acute or chronic), or 593 (other disorders of kidney and ureter). All were assessed from 1978-2004. We also included participants who indicated in 1990 if they had had chronic kidney failure requiring dialysis or transplant.

***Cirrhosis***

Cirrhosis, as indicated by ICD-8 code 571, was assessed starting with the 1978 questionnaire.

***Congestive Heart Failure***

Physician-diagnosed congestive heart failure was assessed in the 98, 00, 02 and 04 questionnaires.

***Connective Tissue Disease***

Connective tissue disease was defined as any physician-diagnosed rheumatoid arthritis, systemic lupus erythematosus  (assessed since 1976 questionnaire), sarcoidosis, or polymyalgia rheumatica (as indicated by ICD-8 code 135 and 725 respectively, assessed since 1978 questionnaire).

***Depression***

This variable includes any physician-diagnosed depression (assessed in 00, 02, 04 questionnaires) as well as use of SSRIs including Prozac, Zoloft, Paxil, Celexa, and others (assessed from 1996-2004).

***Diabetes***

Diabetes was defined as any physician-diagnosed type 2 diabetes, assessed starting with the 1976 questionnaire

***Dementia/Alzheimer’s***

We defined this variable as any physician-diagnosed Alzheimer’s (assessed in the 94, 96, 00 and 02 questionnaires) dementia (as indicated by ICD-8 code 290, assessed since the 1978 questionnaire), or use of Aricept or Namenda, assessed in 2004 only.

***Emphysema/Asthma***

This variable includes any physician-diagnosed emphysema, chronic bronchitis, or asthma, assessed starting with the 1988 questionnaire.

***Gastrointestinal bleed requiring hospitalization***

Participants were asked in 2004 only whether they have ever had gastrointestinal bleeding that required hospitalization or a transfusion.

***Gout***

Physician-diagnosed gout was assessed starting with the 1976 questionnaire.

***High blood pressure***

Physician-diagnosed high blood pressure was assessed starting with the 1976 questionnaire.

***Hip fracture***

Physician-diagnosed hip fracture was assessed starting with the 1992 questionnaire.

***Hypothyroidism***

Physician-diagnosed hypothyroidism was assessed in the 2002 questionnaire only.

***Hyperthyroidism***

Physician-diagnosed hyperthyroidism/Graves’ Disease was assessed in the 2002 and 2004 questionnaires only.

***Inflammatory Bowel Disease***

Physician-diagnosed inflammatory bowel disease was assessed starting with the 1976 questionnaire.

***Mild Liver Disease***

For mild liver disease we included chronic hepatitis, indicated by ICD-8 code 573, assessed since the 1978 questionnaire, as well as viral hepatitis as indicated in 2004 only (due to the episodic nature of the disease).

***Multiple Sclerosis***

Physician-diagnosed multiple sclerosis was assessed starting with the 1976 questionnaire.

***Myocardial Infarction***

Physician-diagnosed myocardial infarction was assessed starting with the 1982 questionnaire.

***Osteoarthritis***

Physician-diagnosed osteoarthritis was assessed in the 1996 and 2000 questionnaires only.

***Osteoporosis***

Physician-diagnosed osteoporosis was assessed starting with the 1982 questionnaire. Use of medications for osteoporosis, including Evista, Fosamax, Miacalcin, and Didronel was assessed starting in 1998. For this variable, we considered either a diagnosis or use of medications an indication of osteoporosis.

***Parkinson’s Disease***

Physician-diagnosed Parkinson’s disease was assessed starting with the 1994 questionnaire.

***Peptic Ulcer Disease***

Physician-diagnosed gastric or duodenal ulcer was assessed starting in 1982, until the 2000 questionnaire.

***Peripheral Vascular Disease***

Peripheral vascular disease includes any physician-diagnosed peripheral artery disease or pulmonary embolus, assessed starting in 1976.

***Pneumonia***

Physician-diagnosed x-ray confirmed pneumonia was assessed in the 2004 questionnaire only. Participants were asked in this questionnaire if they had ever had pneumonia.

***Seizures/epilepsy***

Physician-diagnosed seizure/epilepsy was assessed in the 2004 questionnaire only.

***Stroke/Transient Ischemic Attack***

This variable includes stroke, assessed since the 1988 questionnaire, as well as physician-diagnosed transient ischemic attack, assessed since 1990.

***Use of Statins***

Participants were asked in the 2000, 2002 and 2004 questionnaires if they used of statins, including Mevacor, Zocor, Pravachol, Lipitor, other statins and other cholesterol-lowering drugs. Use of any of these drugs indicated in any of the three questionnaires was considered a use of statins.

***Vertebral fracture***

Physician-diagnosed vertebral fracture was assessed starting with the 1976 questionnaire.

***Wrist fracture***

Physician-diagnosed wrist fracture was assessed starting with the 1976 questionnaire.

**Psychosocial:**

***Amount of emotional support 00, 02, 04- None/a little/some, most/all of the time***

Participants were asked in 2000 and 2004 if they could count on anyone to provide them with emotional support none of the time, a little of the time, some of the time, most of the time, or all of the time. We combined none, a little, and some of the time into one category, and most and all of the time into a second category. We used responses from 2004, and if missing, from 2000.

***Anxiety (almost never, sometimes, often/almost always)***

Participants were asked in 2004 how often they feel nervous and restless (almost never, sometimes, often, almost always). We combined often and almost always into one category. In 2000, they were asked if they have been a very nervous person (all of the time, most of the time, a good bit of the time, some of the time, a little of the time, none of the time). Categories from the 2000 question were collapsed into those from the 2004 question (All/most/a good bit of the time collapsed into often/almost always, some of the time into sometimes, and a little/none of the time into almost never). We then used responses from 2004, and if missing, from 2000.

***Emotional problems interfering with social activities– none vs. all/most/some/little of the time***

Participants were asked in 1996 and 2000 how much of the time during the past 4 weeks had their physical health or emotional problems interfered with social activities (all of the time, most of the time, some of the time, a little of the time, none of the time). We combined all, most, some and little into one category vs. none of the time. We used responses from 2000, and if missing, from 1996.

***Number of people providing emotional support***

Participants were asked in 2000 and 2004 how many people they can count on to provide them with emotional support (none, one, two, three or more). We used responses from 2004, and if missing, from 2000.

***Perceived health (Excellent/Very good, Good, Fair/Poor)***

Participants were asked in the 2000 questionnaire how they would describe their health in general with options “excellent”, “very good”, “good”, “fair”, and “poor”. We combined excellent and very good into one level, and combined fair and poor into one level (3 levels total).

**Breast Cancer Risk Factors:**

***Age at first live birth (nulliparous, <25, 25-29, 30+)/parity***

Age at first live birth and parity were assessed in all questionnaires from 1976 to 1984 as separate variables, and were then categorized and combined into one variable with the following categories: Nulliparous, <25 at first birth with 1-2 or unknown number of children, <25 at first birth with 3+ children, 25-29 at first birth with 1-2 or unknown number of children, 25-29 with 3+ children, 30+ at first birth with 1-2 or unknown number of children, 30+ with 3+ children

***Age at menopause (<45, 45-49, 50-54, 55+)***

We used the NHS derived menopause variable which has been cleaned and identifies participants with inconsistent and uncertain age at menopause which we set to unknown. This variable also uses data on reported age at bilateral oophorectomy to determine age at menopause and the age at which women that underwent simple hysterectomies developed symptoms. The derived variable is continuous but we categorized age at menopause as <45, 45-49, 50-54, 55+.

***First degree relatives with history of breast cancer (0, 1, 2+) and their age at diagnosis***

Whether a mother or sister(s) were diagnosed with breast cancer was collected every 4 years from 1976 to 2004. Data on whether daughter(s) were diagnosed with breast cancer was collected in 2000 and 2004. We categorized participants first by the number of first degree relatives with breast cancer history and then by their relatives’ age at diagnosis. Women who did not know their relatives’ age at diagnosis were grouped with women whose relatives were 50 years or older at diagnosis (lower risk group). The final groups were: None, 1 diagnosed before age 50, 1 diagnosed at 50 or older or unknown age, 2+ with at least 1 diagnosed before age 50, 2+ with 2+ diagnosed at 50 or older or unknown age.

***Mammogram in the past 2 years***

In 2004, the NHS asked participants if they had a mammogram in the past 2 years. If so, a participant reported whether the mammogram was performed for screening or due to symptoms. We considered a woman to have undergone a mammogram if it was screening purposes only. Participants that completed the short version of the 2004 questionnaire were not asked to report on mammography use. If a participant was missing data on mammography use in 2004, we used her response in 2002. If she was missing data on mammography use in both years then we recorded her response as missing.

***Number of Breast Biopsies (0,1,2+)***

We captured biopsies reported from the 1982 through 2004 questionnaires. Each questionnaire asks if participants were diagnosed with benign breast disease since the last questionnaire and if so when and whether the benign breast disease was confirmed by breast biopsy. If a participant reported never having benign breast disease then they were categorized as never having a breast biopsy. If a participant reported having benign breast disease but it was never confirmed by biopsy then we categorized this participant as not having a breast biopsy.

The 1982 and 1984 questionnaires asked the month and year benign breast disease was diagnosed. From 1986 through 2004, questionnaires categorized date of biopsy confirmed benign breast disease as: before June of the first questionnaire year (e.g. before June 1, 2002), during the two questionnaire years (e.g., June 2002 to May 2004), and after June of the second questionnaire year (e.g., after June 2004). We considered biopsies reported during the current questionnaire period as a biopsy performed during that questionnaire period and we tallied the number of biopsies reported.

Biopsies labeled as being done before or after the questionnaire period were assigned as either had a biopsy “before current questionnaire” or “after current questionnaire.” If a participant had biopsy confirmed benign breast disease “before the current questionnaire” followed by biopsy confirmed benign breast disease “after current questionnaire” on a later questionnaire, we categorized these women as having undergone two separate biopsies. If a participant had a biopsy confirmed benign breast disease “before current questionnaire” followed by biopsy confirmed benign breast disease “before current questionnaire” at a later questionnaire, we categorized this biopsy as one biopsy since each of these before responses could be referring to one biopsy performed many years before. If a participant had an “after current questionnaire” followed by a “before current questionnaire” at a later questionnaire, we categorized this biopsy as one biopsy. If a participant had a biopsy after the current time period and then subsequently gave a date during a following questionnaire time period and then gave a “before current questionnaire” in a follow-up questionnaire, we categorized this biopsy as one biopsy. If a participant had a biopsy “after current questionnaire period” followed by a biopsy “after current questionnaire period” we categorized these biopsies as two separate biopsies since a participant must return a questionnaire between these biopsies where she would be able to report that she had a biopsy before the current questionnaire or during the current questionnaire. If a participant reported having a biopsy but never reported the date of the biopsy, then she was categorized as having one biopsy.

Biopsies reported on the 2004 survey were not included if a woman was diagnosed with breast cancer within the first year of follow-up since that biopsy may have been performed as part of diagnosis.

***Menopausal hormone therapy***

Hormone therapy use was assessed beginning in 1978. If a participant never reported using hormone replacement therapy (HRT) we categorized her as having never used HRT. If a woman reported using estrogen plus progesterone (E+P) or estrogen alone (E) on the 2004 questionnaire then we considered her a current user. Women who did not report using HRT on the 2004 questionnaire were categorized as past users and we further categorized these women as past users for <5years or past users for >5 years. NHS records duration of use each time a woman reports use of E+P or E. If a woman reported using postmenopausal hormone therapy but not which type we categorized her as unknown. Since E+P was associated with increased breast cancer risk in the WHI trial while E alone was not, if a woman reported both E+P and E alone use ever she was classified by her E+P use.

**Mammographic Density (predicted):**

The following variables were used to predict mammographic density:

***Age (median-centered)***

We measured age at 2004 questionnaire return date by calculating the number of months between a participant’s birth month and year and month and year of return of the 2004 questionnaire. The number of months was divided by 12 to determine age in years. We used a median-centered age to calculate predicted breast density, based on a median of 69 years.

***Body Mass Index (BMI) (kg/m^2^)***

To calculate BMI, we used the highest self-reported weight within the 10 years before start of follow up. Height was self-reported in 1976. Weight has been assessed on every questionnaire since 1976. We chose to use the highest weight reported in the past 10 years since older women tend to lose weight as they approach death as a result of illness and frailty. We used a median-centered BMI to calculate predicted breast density, based on a median of 26 kg/m^2^.

***Body Mass Index (BMI) at age 18 (kg/m^2^)***

BMI at age 18 is a derived variable created by NHS calculated from participants’ self-reported height and weight at age 18. We used a median-centered BMI to calculate predicted breast density, based on a median of 21 kg/m^2^.

***Adolescent somatotype***

In the 1988 questionnaire, participants were given a set of 9 different figures and were asked to identify which best depicts their outline at ages 5, 10, 20, 30, 40, and currently. Values were treated as numeric ranging from 1 to 9. To determine adolescent somatotype, we took the average of the values given for age 10 and age 20.

***Parity***

Parity was determined from a derived variable for parity created by NHS, most recently updated in 1996, ranging from 0-16 children. This was treated as a continuous variable in our breast density prediction calculation.

***Age at first birth***

Age at first birth was determined from a derived variable created by NHS, ranging from 14-47. We used a median-centered age at first birth to calculate breast density, based on a median of 25 years. If a participant was nulliparous, they were given a value of 25 years, or a median-centered 0 years.

***Nulliparous***

This is a dichotomous variable based on parity. If a participant had 0 children according to the 1996 derived parity variable, they were considered nulliparous. Otherwise, they were not considered nulliparous.

***Benign breast disease (biopsy unconfirmed)***

Starting with the 1976 questionnaire, participants were asked if they had had clinician-diagnosed fibrocystic/other benign breast disease. Any participant that indicated a yes to this variable between 1976 and 2004 was included.

***Benign breast disease (biopsy confirmed)***

Starting with the 1982 questionnaire, participants were asked if they had had clinician-diagnosed fibrocystic/other benign breast disease confirmed by breast biopsy. Any participant that indicated confirmation by breast biopsy between 1982 and 2004 was included.

***Hormone therapy use (Never, Past, Current)***

Hormone therapy use was assessed beginning in 1978. If a participant never reported using hormone replacement therapy (HRT) we categorized her as having never used HRT. If a woman reported using estrogen plus progesterone (E+P) or estrogen alone (E) on the 2004 questionnaire then we considered her a current user. Women who did not report using HRT on the 2004 questionnaire were categorized as past users. For the purpose of the predicted breast density calculation we used the categories “Never”, “Past” and “Current”.

**Other Breast Cancer Risk Factors Considered (but not included in the final model).**

***Age at menarche (</=11, 12-13, 14+)***

Assessed in the 1976 questionnaire. Range in ages 6-49. We recoded outliers >21 years as missing observations.

***Ashkenazi Jewish***

Religious heritage was assessed in 1996. Participants were asked if they were Ashkenazi Jewish. We categorized women as yes/no/missing.

***Family history of ovarian cancer***

History of ovarian cancer in a mother or sister(s) was assessed every four years beginning in 1992 and in a daughter in 2004. We categorized responses as one or more fist degree relative with ovarian cancer versus none.

***Grandmother with history of breast cancer***

History of breast cancer in a maternal and/or paternal grandmother was assessed in 1988. NHS did not assess the age of the grandmother at diagnosis. We categorized responses as one or more grandmother with breast cancer versus none. NHS did not assess history of breast cancer in other second or third degree relatives.

***Number of months breastfeeding(0, <12, 12-23, 24+)***

Participants were asked on the 1986 questionnaire how many months they have breastfed in total. We categorized the variable into none, less than 12 months, 12 to less than 24 months, and 24+ months.

**Black Women’s Health Study (BWHS):**

***Breast cancer***

Incident invasive breast cancer cases were identified through self-report on biennial questionnaires or through 24 state cancer registries in states in which >95% of BWHS participants live, and the diagnoses were confirmed by review of hospital and state cancer registry pathology records.

***Death***

Deaths were determined from state-issued death certificates and the National Death Index. Deaths were counted if they occurred between the 2009 questionnaire return date and 10 years following that date. Deaths were confirmed through 2020. We censored women at the end of 2020 if their 10-year follow-up ended after that.

***Breast cancer death/ Non-breast cancer death***

Cause of death was determined from state-issued death certificates and the National Death Index. In each follow-up cycle, the National Death Index was searched for study participants who did not complete that questionnaire. Women were classified as having died from breast cancer if breast cancer was listed as an immediate or underlying cause of death. Otherwise, a death was considered a non-breast cancer death.

***Age***

Age was reported on the 2009 questionnaire.

***Body Mass Index (BMI) (kg/m^2^)***

BMI was calculated for each questionnaire using reported height and weight. We used the highest BMI from the past 10 years.

***Alcohol consumption, maximum gram/day in the past 10 years (none, 1-<5 gm/day, 5-<15 gm/day, 15+ gm/day)***

We used alcohol consumption data that were collected between the1999-2009 questionnaires.

We took the maximum value of alcoholic beverages per week in this 10 year period and converted it to grams per day, using a conversion rate of 14 grams per alcoholic beverage. We divided these values into 4 categories consistent with NHS.

***Cigarette use (never, past, current)***

Cigarette use was collected on every questionnaire. For each questionnaire through 2009, if a participant reported smoking > 0 cigarettes or reported smoking menthol cigarettes they were considered a current smoker in that cycle. We categorized women who used cigarettes on earlier questionnaires, but reported no smoking on more recent questionnaires, as past users. Women who reported no cigarette use across all surveys were considered never smokers. Women who reported cigarette use on their most recent questionnaire were considered current smokers.

***Walking pace***

Participants were asked for their usual walking pace in the 2005 questionnaire. Response categories included casual or strolling, average or normal, fairly brisk, and brisk or striding. We collapsed casual or strolling and average or normal into the slow/average category, and fairly brisk and brisk or striding into the brisk/very brisk category. Participants were not asked whether they were unable to walk.

***High blood pressure***

Physician-diagnosed high blood pressure was assessed starting with the 1995 questionnaire.

***Depression***

Physician-diagnosed depression treated with medication was assessed starting with the 1999 questionnaire.

***Stroke/Transient Ischemic Attach***

Physician-diagnosed stroke was assessed starting with the 1997 questionnaire.

***Hip fracture***

Physician-diagnosed hip fracture was assessed starting with the 2011 questionnaire, as well as in the “other serious illness” write-in field starting with the 1995 questionnaire.

***Parkinson’s***

Physician-diagnosed Parkinson’s disease was assessed from the “other serious illness” write-in field starting with the 1995 questionnaire.

***Myocardial Infarction***

Physician-diagnosed myocardial infarction was assessed starting with the 1995 questionnaire.

***Congestive heart failure***

Physician-diagnosed stroke was assessed starting with the 2007 questionnaire.

***Stroke***

Physician-diagnosed stroke was assessed starting with the 1995 questionnaire.

.

***Emphysema/Asthma***

Physician-diagnosed asthma was assessed starting with the 1997 questionnaire. Emphysema and chronic obstructive pulmonary disease (COPD) were assessed from the “other serious illness” write-in field starting with the 1995 questionnaire. COPD was also queried explicitly on the 2019 questionnaire.

***Diabetes***

Physician-diagnosed stroke was assessed starting with the 1997 questionnaire.

***Dementia***

Alzheimer’s disease and dementia were queried explicitly in the 2017 and 2019 questionnaires. They were also assessed from the “other serious illness” write-in field starting with the 1995 questionnaire.

***Kidney Disease***

Physician-diagnosed end-stage renal disease was assessed starting with the 2007 questionnaire.

***Cancer***

Cancer was assessed starting with the 1995 questionnaire. All cancers except non-melanoma skin cancer and breast cancer were included.

***Age at menopause (<45, 45-49, 50-54, 55+)***

We used the BWHS derived menopause variable which uses extensive logic to clean menopause data, taking into account hysterectomy, oophorectomy, age and other special conditions. 22% of women had had a hysterectomy with an unknown age, which were given their own category. For analyses we assigned these women to the 45-49 category (after performing sensitivity analyses which showed no significant difference when placed in any other category).

***Mammogram in the past 2 years***

If a participant reported on the 2009 questionnaire that she had a mammogram since March 2007, she was categorized as having undergone mammography.

***Number of Breast Biopsies (0,1,2+)***

In the 2009 questionnaire participants were asked how many breast biopsies they had ever had. We categorized them as having had 0, 1 or 2+ biopsies.

***Menopausal hormone therapy***

Hormone therapy use was assessed beginning with the 1995 questionnaire. If a participant never reported using hormone replacement therapy (HRT) we categorized her as having never used HRT. If a woman reported using estrogen plus progesterone (E+P) or estrogen alone (E) on the 2009 questionnaire then we considered her a current user, and was further categorized by duration of use: <5 years or >5 years .Women who did not report using HRT on the 2009 questionnaire, but reported it previously were categorized as past users and we further categorized these women as past users for <5years or past users for >5 years. BWHS records duration of use each time a woman reports use of E+P or E. If a woman reported using postmenopausal hormone therapy but not which type we categorized her as unknown.

***Age at first live birth (nulliparous, <25, 25-29, >30]/parity***

Age at first live birth and party were assessed as separate variables starting with the 1995 questionnaire and were then categorized and combined into one variable with the following categories: Nulliparous, <25 at first birth with 1-2 or unknown number of children, <25 at first birth with 3+ children, 25-29 at first birth with 1-2 or unknown number of children, 25-29 with 3+ children, 30+ at first birth with 1-2 or unknown number of children, 30+ with 3+ children.

***First degree relatives with history of breast cancer (0, 1, 2+) and their age at diagnosis***

Whether a mother or sister(s) were diagnosed with breast cancer was collected in the 1995 and 1999 questionnaires. Whether or not they were diagnosed before age 50 was collected in the 1995 questionnaire only. We categorized participants first by the number of first degree relatives with breast cancer history and then by their relatives’ age at diagnosis. Women who did not know their relatives’ age at diagnosis were grouped with women whose relatives were 50 years or older at diagnosis (lower risk group). The final groups were: None, 1 diagnosed before age 50, 1 diagnosed at 50 or older or unknown age, 2+ with at least 1 diagnosed before age 50, 2+ with 2+ diagnosed at 50 or older or unknown age.

***Race/ethnicity***

All participants were Black. Ethnicity (Hispanic or non-Hispanic) was assessed on the 1997 questionnaire.

**Appendix C. More Detailed Description of Methods for Multiple Imputation**

More detailed methods on validating the CRR model.

Calibration of the model in predicting non-BC death was assessed by estimating the ratio of the expected survival (1-CIF) for non-BC death to the observed survival (1-CIF) for non-BC at 5 and 10 years within risk quintiles. The expected CIF was computed using CRR and the observed CIF was computed using the nonparametric estimation of CIF (reference: Gray, R. J. (1988). “A Class of K-Sample Tests for Comparing the Cumulative Incidence of a Competing Risk.” *Annals of Statistics* 16:1141–1154). This observed estimate of CIF has the following form:

$$CIF\left( t_{f},nonBC death \right)=\sum_{l=1}^{f} S\left( t_{l-1} \right)\times h(t_{l},nonBC death)\text{ }$$

Where $t_{f}$is the ordered times of nonBC death event, $S\left( t_{l-1} \right)$ is the survival function up to the previous time interval from both nonBC death and competing events (BC death), $h(t_{l},nonBC death)\text{ }$is the hazard estimate in the current time $t_{l}.$

To compute the c-statistic in the validation data, we used the survcstd SAS macro (citation below). The algorithm used in this code is essentially based on the work of Harrell et al. (1996), and later expanded by Pencina et al. (2004). Similar to the concept used for the c-statistics in logistic regression models, the c-index was created for survival analysis models. The concept of concordance is also used to evaluate the discrimination of the model. For the c-index, concordance was based on the idea that for any pair of observed survival times for subject *i^th^* and *j^th^*, t_i_ and t_j_, where t_i <_ t_j_, if the predicted survival times from the model also show the same order or predicted survival times then we have concordance. The ratio of concordant pairs to the total number of eligible pairs of survival times estimates the c-index. However, estimating predicted survival times is equivalent to estimating survival functions S(t) for subject *i^th^* and *j^th^* to reach a survival time *t* where the observed *t_i_ < t_j_ ≤ t*, and the equivalent order of these quantities for concordance is S_i_(t)<S_j_(t). But this inequality is equivalent to S_0_(t)^exp(X^_i_^β)^ < S_0_(t)^exp(X^_j_^β)^ , but S_0_(t), the baseline survival function is between 0 and 1, and thus this is equivalent to X_i_β > X_j_β. In other words, the linear combination of the subject covariates X, and the model estimated coefficients for β is all that is needed to define the concordance or discrimination measure c-index in survival analysis. For our validation, we used the model coefficients β from the development cohort (NHS Fine-Gray model), and the covariates X from the validation dataset. The key input into the macro to generate the c-index is this quantity Xβ, termed “score” in the code.

Kremers, W. K. (2007). Technical Report Series No. 80, Concordance for survival time data: Fixed and time-dependent covariates and possible ties in predictor and time. Department of Health Science Research, Mayo Clinical, Rochester, Minnesota, 2007.

<https://www.mayo.edu/research/documents/biostat-80pdf/doc-10027891>

Harrell FE, Jr., Lee KL, Mark DB. Multivariable prognostic models: issues in developing models, evaluating assumptions and adequacy, and measuring and reducing errors. *Stat Med.* 1996;15(4):361-387.

Pencina MJ, D'Agostino RB. Overall C as a measure of discrimination in survival analysis: model specific population value and confidence interval estimation. *Stat Med.* 2004;23(13):2109-2123.

Multiple Imputation:

Seven variables included in the final model for predicting non-breast cancer death (alcohol use [10% missing], cigarette use [0.2% missing], limited walking [5.4% missing], limited bathing/dressing [5.3% missing], walking pace [4.6% missing], age at menopause [1.0% missing], and mammogram in past two years [8.5% missing]) were missing data. For each of these 7 variables we compared the individuals with missing data to those not missing data by 11 comorbidities. These groups did not differ from one another by > than 6.5%, and most comparisons showed much smaller differences. Also, there was no pattern in the direction of difference. For example, in some comparisons the non-missing group had a higher rate of a specific disease for others that missing group had a higher rate of a specific disease. Thus we concluded the data was missing at random.

We imputed each of these 7 variables using the fully conditional specification method of multiple imputation. Each of the 21 variables in the model were used for imputation, including the dependent variable non-breast cancer death. One hundred imputations were performed. The distribution of the observed and imputed data was similar, as were the hazard ratios between the original and imputed models. The c-statistic of the imputed model was slightly lower than that of the non-imputed model, see eTable 5. Since results were similar with and without multiple imputation we used our non-imputed model in our final analyses.
